# Deep brain stimulation surgical timing, outcomes, and prognostic factors in patients with Parkinson’s disease: A Chinese retrospective multicenter cohort study

**DOI:** 10.1371/journal.pmed.1004670

**Published:** 2025-08-01

**Authors:** Shu Wang, Wei Hu, Yuan Gao, Anxin Wang, Ling Chen, Zhanhua Liang, Shizhong Zhang, Hao Long, Weiguo Li, Chaoshi Niu, Weiguo Liu, Guoen Cai, Yuchen Ji, Joseph Tam, Qin Xu, Anchao Yang, Lin Shi, Hua Zhang, Chunlei Han, Guanyu Zhu, Yutong Bai, Lulu Jiang, Tao Li, Shan Xue, Hongxiao Wang, Yuexuan Li, Chi Xiong, Andres M. Lozano, Adolfo Ramirez-Zamora, Wenbin Zhang, Fangang Meng, Jianguo Zhang, DBS-PDCC2 Collaboration

**Affiliations:** 1 Department of Neurosurgery, Beijing Tiantan Hospital, Capital Medical University, Beijing, China; 2 Beijing Neurosurgical Institute, Beijing Tiantan Hospital, Capital Medical University, Beijing, China; 3 Department of Neurology, Norman Fixel Institute for Neurological Diseases, University of Florida, GainesvilleFlorida, United States of America; 4 China National Clinical Research Center for Neurological Diseases, Beijing Tiantan Hospital, Capital Medical University, Beijing, China; 5 Department of Neurology, The First Affiliated Hospital of Sun Yat-sen University, Guangzhou, Guangdong, China; 6 Department of Neurology, The First Affiliated Hospital of Dalian Medical University, Dalian, Liaoning, China; 7 Department of Neurosurgery, Zhujiang Hospital of Southern Medical University, Guangzhou, Guangdong, China; 8 Department of Neurosurgery, Nanfang Hospital, Southern Medical University, Guangzhou, Guangdong, China; 9 Department of Neurosurgery, Qilu Hospital of Shandong University, Jinan, Shandong, China; 10 Division of Life Sciences and Medicine, Department of Neurosurgery, The First Affiliated Hospital of USTC, University of Science and Technology of China, Hefei, Anhui, China; 11 Department of Neurology, The Affiliated Brain Hospital of Nanjing Medical University, Nanjing, Jiangsu, China; 12 Department of Neurology, Center for Cognitive Neurology, Institute of Clinical Neurology, Fujian Medical University Union Hospital, Fuzhou, Fujian, China; 13 Department of Neurosurgery, The First Affiliated Hospital of Zhengzhou University, Zhengzhou, Henan, China; 14 Division of Neurosurgery and Toronto Western Hospital Research Institute, University Health Network, University of Toronto, Toronto, Ontario, Canada; 15 Department of Functional Neurosurgery, The Affiliated Brain Hospital of Nanjing Medical University, Nanjing, Jiangsu, China; 16 Beijing Key Laboratory of Neurostimulation, Beijing, China; 17 Chinese Institute for Brain Research, Beijing, China; Columbia University, UNITED STATES OF AMERICA

## Abstract

**Background:**

Deep brain stimulation (DBS) has been increasingly introduced for patients with Parkinson’s disease (PD). However, there has been extensive controversy regarding its surgical timing. This study aimed to evaluate surgical outcomes of DBS across different PD durations and identify key prognostic factors.

**Methods and findings:**

In this multicenter cohort study, patients with PD who underwent subthalamic DBS between 1/1/2011 and 12/31/2020 from seven representative Chinese national centers were included. Two-year follow-up data were analyzed, accordingly. These patients were classified into short (<5 years), mid (5−10 years), and long (≥10 years) PD duration groups. Primary assessments included part III of the Movement Disorder Society-sponsored revision of the Unified Parkinson’s Disease Rating Scale (MDS-UPDRS-III) at the off-medicine state, Hamilton Anxiety Rating Scale (HAM-A), Hamilton Depression Rating Scale (HAM-D), and Parkinson Disease Questionnaire-39 (PDQ-39) scales. Relative changes in scores were analyzed for within- and between-group comparisons, and prognostic factors were identified via multivariable linear regression. A total of 1,859 patients were screened, and 1,717 patients (749 females) were included for analysis. Respectively, 141, 978, and 598 patients underwent surgeries after short-, mid-, and long-duration. The scores of the MDS-UPDRS-III (off-medicine), HAM-A, HAM-D, and PDQ-39 significantly improved by 46.7% ± 14.1% (mean difference [MD] 25.1, 95% confidence interval [CI] [24.5, 25.7], *P* < 0.001), 54.4% ± 22.4% (MD 8.0, 95%CI [7.5, 8.5], *P* < 0.001), 43.4% ± 22.6% (MD 6.3, 95%CI [5.8, 6.8], *P* < 0.001), and 47.9% ± 17.8% (MD 28.0, 95%CI [27.0, 29.0], *P* < 0.001), respectively, and all the study groups achieved significant improvements (all *P* < 0.001). Notably, patients with mid-PD duration achieved greatest improvements in motor outcomes (versus short: MD 8.0%, 95%CI [4.7%, 11.3%], *P* = 0.008; versus long: MD 5.6%, 95%CI [2.8%, 9.4%], *P* = 0.01), neuropsychological evaluations (anxiety, versus long: MD 15.2%, 95%CI [12.3%, 18.1%], *P* = 0.002; depression, versus long: MD 19.1%, 95%CI [15.6%, 22.6%], *P* < 0.001), and quality of life (versus long: MD 7.6%, 95%CI [5.2%, 10.0%], *P* = 0.007). Levodopa response (short: adjusted *β* 0.42, 95% CI [0.30, 0.54], *P* < 0.001; mid: adjusted *β* 0.17, 95% CI [0.12, 0.22], *P* < 0.001; long: adjusted *β* 0.20, 95% CI [0.12, 0.28], *P* < 0.001) was a unified positive factor of motor response for all three groups. Higher MDS-UPDRS-III (off-medicine) scores (mid: adjusted *β* 0.10, 95% CI [0.05, 0.15], *P* < 0.001; long: adjusted *β* 0.30, 95% CI [0.23, 0.38], *P* < 0.001) were positively correlated with motor outcomes for the mid- and long-duration groups. Nevertheless, it was a negative factor for the short duration group (adjusted *β* −0.25, 95% CI [−0.36, −0.14], *P* < 0.001). The main limitation of this study is the nonrandomized observational nature introduced potential selection bias and imbalanced comparisons.

**Conclusions:**

DBS significantly improved motor, neuropsychological, and quality-of-life outcomes across all PD durations, with the most substantial benefits observed in mid-duration (5–10 years) patients. While levodopa response was a consistent positive prognostic factor for motor response, caution is warranted for short-duration patients with rapidly progressive motor symptoms, as they exhibited less favorable outcomes.

## Author summary

**Why was this study done?:**

- Deep brain stimulation (DBS) has been increasingly introduced for patients with Parkinson’s disease (PD).
- Increasing enthusiasm has been raised in advocating DBS for patients with shorter duration of PD, but there has been extensive controversy regarding its surgical timing.
- Limited studies have compared patients underwent DBS at different PD durations, leaving the potential optimal surgical timing unclear.

**What did the researchers do and find?:**

- A total of 1,717 patients with PD who underwent subthalamic DBS between 1/1/2011 and 12/31/2020 from seven representative national centers were included and classified into short (< 5 years, *n*=141), mid (5–10 years, *n*=978), and long (≥10 years, *n*=598) PD duration groups; and their two-year follow-up data were collected and analyzed.
- DBS significantly improved motor, mood, and quality-of-life outcomes across all PD durations, with the most substantial benefits observed in mid-duration (5–10 years) patients.
- Greater levodopa response suggested better motor improvement, regardless of disease duration, whereas higher baseline motor severity predicted better outcomes in mid-/long-duration PD but poorer outcomes in short-duration PD.

**What do these findings mean?:**

- DBS significantly improved motor function, mood, and quality of life across all PD durations and mid-duration (5–10) might be the optimal surgical timing window.
- While levodopa response predicted motor improvement in all groups, short-duration patients with rapid symptom progression showed reduced benefits.
- The main limitation is that as an observational study, inherent differences between patient groups may affect outcomes, necessitating prospective studies with random patient assignment.

## Abstract 3

To investigate the optimal timing of deep brain stimulation surgery, Shu Wang and colleagues collect data on motor outcomes, neuropsychological evaluations, and quality of life from a large-scale, multicenter cohort of patients with Parkinson's disease.

## Introduction

Parkinson’s disease (PD) is the second most common neurodegenerative disease with increased prevalence [1,2], causing enormous burdens worldwide [3]. Although dopaminergic medication provides initial relief, fluctuations in medication responses and levodopa-induced complications can occur after disease progression within a few years [4,5], which causes functional decline and contributes to a significant loss of quality of life [6]. For these patients, deep brain stimulation (DBS) has shown great potential in achieving better control of symptoms [7–10]. In early clinical practice and studies, DBS was typically deferred for an extended duration of PD [11]. Several randomized clinical trials (RCTs) [12–16] have provided strong evidence that subthalamic deep brain stimulation (STN-DBS) is superior in terms of outcomes than best medical therapy alone for long-term advanced PD [8]. Recently, several studies have suggested that early DBS can also be efficacious and feasible [17–19]. The EARLYSTIM trial enrolled patients with PD with early fluctuations (mean PD duration as 7.5 years) and revealed its benefits in terms of motor symptoms and quality of life over standard medical therapy [20]. With these encouraging results, stepwise approvals for DBS in patients with advanced (since 2002) to earlier (since 2016, at least 4 years of PD and inadequate medication control) PD have been granted by the U.S. Food and Drug Administration (FDA) [8,21].

However, there is extensive controversy regarding surgical timing [8,22–25]. While some studies have suggested the potential to better enhance quality of life and activities of daily living when DBS is applied early, others are concerned with its uncertain benefits and potential risks [11,22,23,25,26]. To date, only limited evidence is available for the effects of early DBS for PD, especially for patients with very short durations (1–4 years) [11]. In addition, the potential optimal surgical timing could not be determined until patients underwent DBS at different disease durations, including in the same study for comparison [18,23]. To bridge this critical research gap, we collected comprehensive motor outcomes, neuropsychological evaluations, and quality of life data from a large-scale multicenter cohort of patients with PD (1,717 individuals), thus far available for analysis so that DBS performed at different disease durations can be compared to determine the potentially optimal timing for surgery [22]. In addition, we identified several key prognostic factors and their crucial variations, which could help further improve individual outcomes [27,28].

## Methods

### Study design

We performed a nationwide multicenter retrospective cohort study (DBS-PD Chinese Collaboration-2) on STN-DBS for patients with PD with different disease durations from 7 representative university centers between 1/1/2011 and 12/31/2020. This study was approved by the ethics committee of Beijing Tiantan Hospital, Capital Medical University, and all participating centers (KY2021-159-01). An authoritative third party, the China National Clinical Research Center for Neurological Diseases (NCRCND), supervised this study. All included participants provided written informed consent for the surgery and the use of deidentified data for scientific research. This study was performed under the Declaration of Helsinki and was reported as per the Strengthening the Reporting of Observational Studies in Epidemiology (STROBE) guideline (S1 Checklist) [29].

On the basis of our previous study regarding the utilization of DBS for PD (DBS-PDCC) [30], we collected data from well-known experienced national DBS centers (S1 Table) for representatives with the assistance from the Chinese Neuromodulation Society [31,32]. Each participating center submitted case report forms. The study committee, which consists of senior neurologists and neurosurgeons, further reviewed these forms, and each center for all data collection. All deidentified data were included in our DBS-PDCC databases [33,34]. Experts in statistics performed data control, analysis, and validation in a blinded manner. The data collection was finished by December 2023, and the data analyses were conducted from January 2024 to May 2024.

### Participants

At the time of surgery, all patients have received multidisciplinary assessments and fulfilled the surgical indications of DBS for PD [8,16,35] as follows: (1) idiopathic PD diagnosis on the basis of the Movement Disorders Society Clinical Diagnostic Criteria [36] and the UK Brain criteria [37]; (2) the presence of disabling motor complications or levodopa-induced dyskinesia despite receiving optimal medications; (3) the absence of dementia and other clinically relevant cognitive impairment, ongoing severe psychiatric diseases, and severe brain atrophy; and (4) confirmation for surgical need after carefully considering patients’ willing, expected benefits and risks, and their fitness for surgery without systemic contraindications [8,10], especially for older patients aged ≥75 years [8,38,39]. Patients were eligible for the study if they met the following inclusion criteria: (1) were aged 18 years or older; (2) received bilateral STN-DBS at the seven centers from 2011 to 2020; and (3) finished no less than 2 years of follow-up. Patients without written informed consent or with freezing of gait) were excluded. For patients with inadequate follow-up periods or missing data, the research committee first tried to contact the patients and their relatives for further interviews, and those who could not be contacted were excluded.

**Fig 1:**
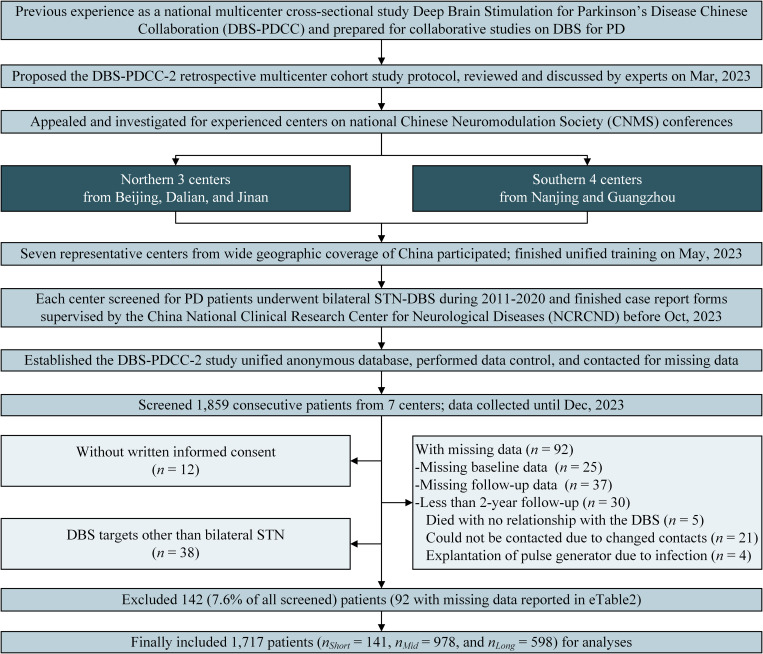
Study flow and data acquisition. DBS-PDCC, Deep Brain Stimulation for Parkinson’s Disease Chinese Collaboration; CNMS, Chinese Neuromodulation Society; PD, Parkinson’s disease; STN-DBS, subthalamic nucleus deep brain stimulation; NCRCND, China National Clinical Research Center for Neurological Disease.

A total of 1,859 patients were screened for eligibility; 142 patients (7.6%) were excluded because they did not provide informed consent (*n* = 12), DBS at targets other than the bilateral STN (*n* = 38), or had missing data (*n* = 92). The details of the noneligible samples are summarized in S2 Table. Finally, this study analyzed 1,717 patients (Beijing 1 center included 725 patients, Liaoning 1 center included 113 patients, Shandong 1 center included 313 patients, Jiangsu 1 center included 370 patients, and Guangdong 3 centers included 196 patients), which were then grouped into short (<5 years), mid (5–10 years), and long (≥10 years) PD duration groups based on FDA stepwise approvals [19,21] and clinical practice in early DBS surgeries [11,23]. The study flow is shown in Fig 1.

### Surgical procedures

Each center performed bilateral STN-DBS surgeries independently for patients with PD. Our interviews suggested that their operative routine and principles were similar to those of our previous studies [40,41]. Generally, preoperative MR images are merged with CT scans after stereotactic frame fixation to visually target the STN and determine the trajectory for electrode placement. After that, the stimulation electrodes (Medtronic-3389, Medtronic Plc; or PINS-L301, PINS Medical; with the same design as the quadripolar contact length of 1.5 mm and spaced 0.5 mm apart) were implemented with further refinement via microelectrode recordings and intraoperative testing for clinical response under local anesthesia.

After the surgery, the lead position and potential complications were evaluated with postoperative neuroimaging, and further test stimulation was evaluated. A few days later, the pulse generator (Activa RC, Medtronic Plc; or Model G102R, PINS Medical) was connected to the intracranial leads and positioned in the subcutaneous region under general anesthesia. The stimulator was initiated 1 month later [34], and a specialized programming team adjusted the stimulation parameters in the follow-ups as needed according to individual symptoms.

### Outcome measurements

Preoperative characteristics were recorded. Young-onset PD was defined as the onset of symptoms before 40 years of age [42,43]. At (preoperative) baseline, at 6 months and every year postoperatively, patients were evaluated by experienced neuropsychologists at their local centers. All the assessment scales used were validated Chinese versions. For preoperative motor assessments, the clinically defined “off-medicine” and “on-medicine” states [10,27,44] were assessed by movement disorder specialists under conditions of no medication effects (at least medication withdrawal for 12 h) and maximal benefits (approximately 1 h after medication intake), respectively. Postoperative outcomes were evaluated at the “on-stimulation” status (at least a 1-week observation period after continuous stimulation without changes in parameters) and the abovementioned “off-medicine” and “on-medicine” conditions [10,27]. To ensure accuracy and quality control, all original measurements were further reviewed by the study committee.

### Primary outcomes

On the basis of assessment tools used at each center, we prospectively selected unified measurements for primary and secondary motor outcomes, neuropsychological evaluations, and quality of life. The outcomes were analyzed at the 2-year follow-up. The Movement Disorder Society-sponsored revision of the Unified Parkinson’s Disease Rating Scale (MDS-UPDRS; four parts, I, II, III, and IV) [45] was used to assess the severity of PD symptoms [46]. Part III of the MDS-UPDRS (MDS-UPDRS-III) [47] in the “off-medicine” state was assessed as the primary motor outcome. The Hamilton Anxiety Scale (HAM-A) [48] and the Hamilton Depression Scale (HAM-D) [49] were used to evaluate anxiety and depression symptoms, respectively. In addition, the Parkinson Disease Questionnaire-39 (PDQ-39) [50] was used to assess quality of life.

### Secondary outcomes

The secondary outcomes for motor measures included part II (MDS-UPDRS-II), part III at the “on-medicine” state, and part IV (MDS-UPDRS-IV) [45] of the MDS-UPDRS; the levodopa equivalent daily dose (LEDD) [51,52]; and the patient motor diary, which includes daily “off-medicine” time (off-time) and “on-medicine” time with troublesome dyskinesia (dyskinesia-time) [53]. Patients were also evaluated with the Hoehn and Yahr scale in the “off-medicine” state [54] and with the levodopa responsiveness score (calculated as a percentage of the MDS-UPDRS-III “off-medicine” score minus the “on-medicine” score divided by the MDS-UPDRS-III “off-medicine” score) [55] at baseline. All motor tests were videotaped with available informed consent. Part I of the MDS-UPDRS (MDS-UPDRS-I) was assessed for nonmotor symptoms. The Mini-Mental Status Exam (MMSE) and the Montreal Cognitive Assessment (MoCA) were also evaluated, whereas lower scores indicate greater cognitive impairment. Any adverse events (AEs) related to the surgery, stimulation, or device were recorded and analyzed as the number of patients and events.

### Statistical analysis

The statistical analysis was planned prospectively (S1 File) in designing the study without deviations. Because of the potential inclusion of important variables in the regression analysis and the low percentage of missing data (4.9%), complete data analysis (listwise deletion) was applied without imputation [56]. We also compared samples with missing data to the samples included and found comparable characteristics and outcomes, suggesting its limited influence (S2 Table).

The Kolmogorov‒Smirnov test and Levene’s test were performed for normality and homogeneity of variance, respectively. Continuous variables were presented as means (± standard deviations, SDs, for normal distribution) or medians (interquartile ranges, IQRs, for nonnormal distribution), whereas categorical variables were reported as numbers (percentages). For within-group comparisons in exploring treatment effects, postoperative scores at 24 months were compared with the baseline using two paired-sample *t* tests. For between-group comparisons, considering imbalance in baseline assessments among different PD duration groups, we calculated improvement percentages (relative changes, [preoperative minus postoperative score]/preoperative score × 100%, for higher scores indicating more severe symptoms, such as the MDS-UPDRS; or its inverse for lower scores indicating more severe symptoms, such as the MMSE and MoCA) for each patient and assessed statistical differences using one-way ANOVA, with pairwise multiple comparisons adjusted by Bonferroni method (multiplied corrected *P* values by three times). All within-group (for scores, postoperative minus preoperative) and between-group (for differences in improvement percentages) comparisons were reported with mean difference (MD) and confidence interval (CI). Correlation analysis was performed using Pearson’s correlation coefficient.

Potential prognostic factors were identified using multivariable linear regression. Independent variables showing *P* < 0.10 in univariable analysis were included in multivariable backward elimination modeling after confirming no significant multicollinearity (variance inflation factors < 10). Final model presented adjusted *β* coefficients with 95% CI. To make it more intuitive, cognitive impairment measured by MMSE and MoCA was operationalized by reverse-coding their original scores (e.g., impairments in MoCA = -MoCA), where higher values now indicate greater cognitive impairment. In regression models, a negative *β* coefficient for “impairment in MoCA” signifies worse outcomes with increasing cognitive impairment.

All the statistical tests were two-sided, and a *P* value (or Bonferroni corrected *P* value) < 0.05 was considered statistically significant. All the statistical analyses and figures were generated via SPSS software, version 27 (IBM Corp.), R software, version 4.3 (R Foundation), and GraphPad Prism, version 10 (GraphPad Software LLC).

## Results

### Baseline characteristics

**Fig 2:**
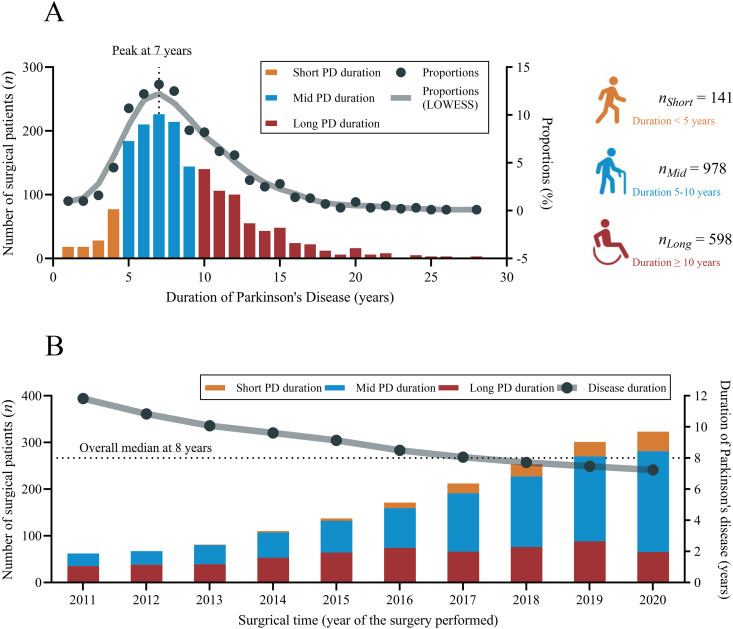
Study groups, distributions, and time trends of included patients. **A**: study groups and their distributions; **B**: time trends of median PD duration at the surgery and changes in study group distributions with time. PD, Parkinson’s disease; LOWESS, locally weighted scatterplot smoothing.

A total of 1,717 patients (749 females, 43.6%) with PD were included from 7 representative specialized DBS centers, of whom 141, 978, and 598 patients underwent DBS after short, mid, and long disease duration, respectively. The study centers and number of patients included at each center are shown in S1 Fig. As shown in Fig 2, most of the patients (57.0%) underwent the surgery after mid PD duration, and the peak disease duration at surgery was 7 years. During the study period, the proportions of mid- and short-duration groups increased, and the yearly median disease duration gradually decreased, with an overall median of 8 years.

Table 1 summarizes the characteristics of the participants. The surgical age was significantly greater in the long duration group than the mid-group, which in turn were higher than in the short duration group (66.0, IQR [60.0–70.0] versus 63.0, IQR [56.0–68.0] versus 58.0, IQR [51.0–63.5]; respectively; all *P* < 0.001). Compared with the short- and mid-duration groups, the long PD duration group presented more severe symptoms on the MDS-UPDRS-III (off-medicine), MDS-UPDRS-II, LEDD, off-time, HAM-A, and HAM-D, whereas the short- and mid-duration groups were comparable. The PDQ-39 scores were significantly different among the 3 groups (64.5, [±17.2] versus 56.4 [±17.3] versus 49.7 [±16.2] for the short-, mid-, and long-duration groups, respectively; all *P* < 0.001).

**Table 1:** Baseline characteristics and assessments of the included patients with Parkinson’s disease (PD) who underwent subthalamic nucleus deep brain stimulation (STN-DBS).

Variable	No. (%); Mean (±standard deviation, SD); Median (interquartile range, IQR)				*P*
	Total (*n *= 1,717)	Short PD duration (*n *= 141)	Mid PD duration (*n *= 978)	Long PD duration (*n *= 598)	
**Characteristic**					
Sex					
Male	968 (56.4%)	79 (56.0%)	555 (56.7%)	334 (55.9%)	0.94
Female	749 (43.6%)	62 (44.0%)	423 (43.3%)	264 (44.1%)	
Age at surgery, year	64.0 (57.0, 69.0)	58.0 (51.0, 63.5)^a^	63.0 (56.0, 68.0)^b^	66.0 (60.0, 70.0)^c^	<0.001^*^
Disease duration, year	8.0 (6.0, 11.0)	4.0 (2.0, 4.0)^a^	7.0 (6.0, 8.0)^b^	12.0 (11.0, 15.0)^c^	<0.001^*^
Age at onset, year	55.0 (48.0, 60.0)	54.0 (47.5, 61.0)	56.0 (49.0, 61.0)	55.0 (48.0, 60.5)	0.31
Young onset PD					
Yes	124 (7.2%)	17 (12.1%)	66 (6.7%)	41 (6.9%)	0.07
No	1,593 (92.8%)	124 (87.9%)	912 (93.3%)	557 (93.1%)	
Motor fluctuations					
Yes	1,717 (100.0%)	141 (100.0%)	978 (100.0%)	598 (100.0%)	NA
Dyskinesia					
Yes	1,069 (61.2%)	77 (54.6%)	602 (61.6%)	390 (65.2%)	0.051
No	678 (38.8%)	64 (45.4%)	376 (38.4%)	208 (34.8%)	
Hoehn and Yahr stage (off-medicine)	3.0 (±0.6)	2.7 (±0.6)^a^	2.9 (±0.6)^b^	3.2 (±0.7)^c^	<0.001^*^
**Assessment**					
Motor measure					
MDS-UPDRS-III (off-medicine, motor)	53.8 (±13.1)	49.8 (±14.7)	53.9 (±12.9)^b^	54.6 (±13.5)^c^	<0.001^*^
MDS-UPDRS-III (on-medicine, motor)	25.8 (±6.8)	22.3 (±8.1)	24.6 (±6.4)	28.5 (±7.2)	0.14
Levodopa responsiveness, %	52.0 (±7.9)	55.2 (±9.1)	54.4 (±7.3)^b^	47.8 (±8.6)^c^	<0.001^*^
MDS-UPDRS-II (daily living)	20.3 (±6.2)	18.4 (±6.1)	19.8 (±6.0)^b^	21.6 (±6.4)^c^	<0.001^*^
MDS-UPDRS-IV (complications)	7.3 (±3.6)	5.5 (±2.3)	6.9 (±3.0)	8.0 (±4.2)	0.10
Levodopa-equivalent daily dose, mg	776.5 (±349.9)	681.7 (±269.8)	738.7 (±312.2)^b^	861.9 (±385.6)^c^	<0.001^*^
Patient motor diary					
Off time, h/d	5.8 (±1.7)	5.1 (±1.6)	5.6 (±1.7)^b^	6.8 (±1.9)^c^	<0.001^*^
On time with troublesome dyskinesia, h/d	4.0 (±1.9)	3.5 (±1.9)	3.9 (±1.8)	4.3 (±2.1)	0.06
**Neuropsychological evaluation**					
HAM-A	14.7 (±8.6)	13.3 (±8.7)	13.6 (±8.3)^b^	16.9 (±8.7)^c^	<0.001^*^
HAM-D	14.5 (±8.1)	13.8 (±8.3)	14.0 (±8.1)^b^	15.7 (±8.0)^c^	<0.001^*^
MDS-UPDRS-I (non-motor experiences)	14.3 (±4.6)	13.4 (±5.3)	14.3 (±3.9)	16.0 (±5.1)	0.25
MMSE	26.4 (±4.9)	27.2 (±4.8)	26.6 (±4.9)	25.3 (±5.8)	0.11
MoCA	25.8 (±4.7)	26.5 (±4.7)	25.9 (±4.7)	24.8 (±4.5)	0.09
**Quality of life**					
PDQ-39	58.5 (±17.1)	49.7 (±16.2)^a^	56.4 (±17.3)^b^	64.5 (±17.2)^c^	<0.001^*^

PD, Parkinson’s disease; STN-DBS, subthalamic nucleus deep brain stimulation; SD, standard deviation; IQR, interquartile range (Q1-Q3); MDS-UPDRS, the Movement Disorder Society-sponsored revision of the Unified Parkinson’s Disease Rating Scale (scale part I, II, III, IV); HAM-A, Hamilton Anxiety Rating Scale; HAM-D, Hamilton Depression Rating Scale; MMSE, Mini-Mental Status Examination; MoCA, Montreal Cognitive Assessment; PDQ-39, Parkinson Disease Questionnaire-39; NA, not applicable.

*P*, *P* value of overall comparison for between-group differences (those with significance would be further tested with pairwise analyses).

* *P* < 0.01 (Kruskal-Wallis test or one-way ANOVA, as appropriate).

^a^*P* < 0.05 (multiple comparisons adjusted by Bonferroni method; tests for pairwise comparison of short and mid PD duration groups).

^b^*P* < 0.05 (multiple comparisons adjusted by Bonferroni method; tests for pairwise comparison of mid and long PD duration groups).

^c^*P* < 0.05 (multiple comparisons adjusted by Bonferroni method; tests for pairwise comparison of short and long PD duration groups).

### Primary motor, neuropsychological outcomes, and quality of life

**Fig 3:**
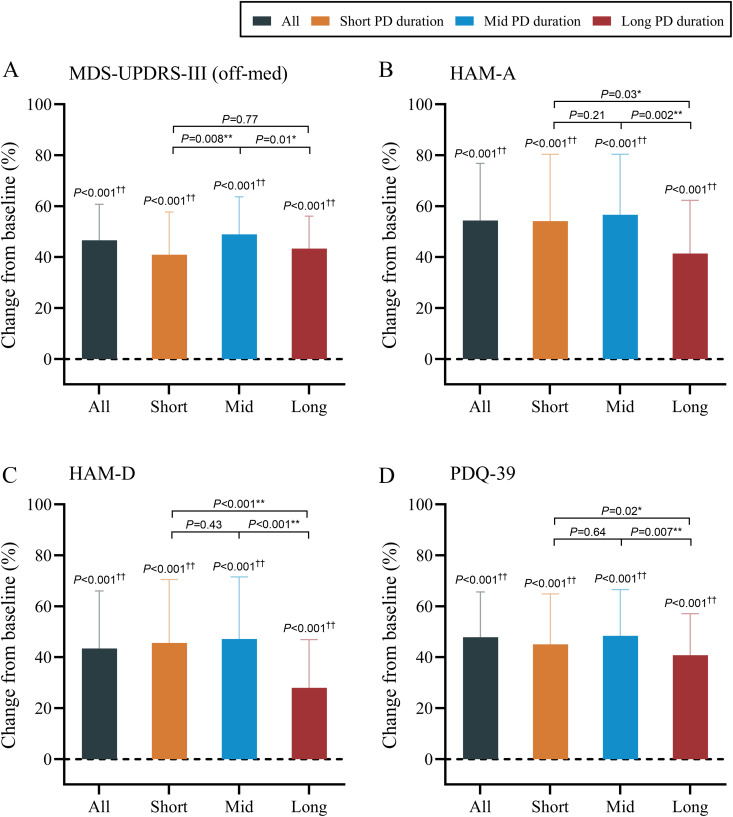
Primary motor outcomes, neuropsychological evaluations, and quality of life at 24-month follow-up after the surgery. **A**: Motor outcomes by MDS-UPDRS-III (off-medicine); **B** and **C**: Neuropsychological evaluations by HAM-A and HAM-D; **D**: quality of life by the PDQ-39. ^†^*P* < 0.05 (within-group comparisons, paired *t t*est); ^††^*P* < 0.01 (within-group comparisons, paired *t* tes*t*); *P < 0.05 (between-group comparisons, one-way ANOVA with multiple comparisons adjusted by Bonferroni method); **P < 0.01 (between-group comparisons, one-way ANOVA with multiple comparisons adjusted by Bonferroni method). The error bars represent the standard deviation (SD). MDS-UPDRS, Movement Disorder Society-sponsored revision of the Unified Parkinson’s Disease Rating Scale (with parts I, II, III, IV); HAM-A, Hamilton Anxiety Rating Scale; HAM-D, Hamilton Depression Rating Scale; PDQ-39, Parkinson Disease Questionnaire-39.

All surgical outcomes are reported in Table 2 (detailed within-group and between-group comparisons are shown in S3 and S4 Tables) and the primary outcomes are shown in Fig 3. In general, motor outcomes measured by the MDS-UPDRS-III (off-medicine) significantly improved from baseline by 46.7% ± 14.1% (MD 25.1, 95% CI [24.5, 25.7], *P* < 0.001). In the neuropsychological evaluations, symptoms of anxiety and depression also significantly improved by 54.4% ± 22.4% (MD 8.0, 95%CI [7.5, 8.5], *P* < 0.001) and 43.4% ± 22.6% (MD 6.3, 95%CI [5.8, 6.8], *P* < 0.001), respectively (all *P* < 0.001), and quality of life, as measured by the PDQ-39, significantly improved by 47.9% ± 17.8% (MD 28.0, 95%CI [27.0, 29.0], *P* < 0.001). All study groups had significant improvements in the MDS-UPDRS-III (off-medicine), HAM-A, HAM-D, and PDQ-39 scores (all *P* < 0.001). Between-group analyses revealed that surgeries performed after mid PD duration improved the MDS-UPDRS-III score (off-medicine) more than those performed after short and long PD duration did (vs short duration: MD 8.0%, 95%CI [4.7%, 11.3%], *P* = 0.008; versus long duration: MD 5.6%, 95%CI [2.8%, 9.4%], *P* = 0.01), whereas the scores of the short and long duration groups were comparable (*P* = 0.77). For the HAM-A, HAM-D, and PDQ-39 scores, the mid and short duration groups all presented greater improvements than did the long duration group (HAM-A: mid versus long: MD 15.2%, 95%CI [12.3%, 18.1%], *P* = 0.002, short versus long: MD 12.7%, 95%CI [7.6%, 17.8%], *P* = 0.03; HAM-D: mid versus long: MD 19.1%, 95%CI [15.6%, 22.6%], *P* < 0.001, short versus long: MD 17.7%, 95%CI [12.5%, 22.9%], *P* < 0.001; PDQ-39: mid versus long: MD 7.6%, 95%CI [5.2%, 10.0%], *P* = 0.007, short versus long: MD 4.3%, 95%CI [0.5%, 8.1%], *P* = 0.02), whereas no significant differences were revealed between the mid and short duration groups (all *P* > 0.05).

**Table 2:** Primary and secondary motor, neuropsychological outcomes, and quality of life of the included patients with Parkinson’s disease (PD) at 24 months after subthalamic nucleus deep brain stimulation (STN-DBS).

Outcome	Mean score (±standard deviation, SD)												
	Total (*n *= 1,717)			Short PD duration (*n *= 141)			Mid PD duration (*n *= 978)			Long PD duration (*n *= 598)			*P*
	Baseline	24 mo	% Improv	Baseline	24 mo	% Improv	Baseline	24 mo	% Improv	Baseline	24 mo	% Improv	
**Primary outcome**													
Motor measure													
MDS-UPDRS-III (off-medicine, motor)	53.8 (±13.1)	28.7 (±12.2)^†^	46.7% (±14.1%)	49.8 (±14.7)	29.4 (±13.9)^†^	41.0% (±16.8%)^a^	53.9 (±12.9)	27.5 (±13.4)^†^	49.0% (±14.7%)^b^	54.6 (±13.5)	30.9 (±11.4)^†^	43.4% (±12.7%)	<0.001^*^
Neuropsychological evaluation													
HAM-A	14.7 (±8.6)	6.7 (±4.0)^†^	54.4% (±22.4%)	13.3 (±8.7)	6.1 (±3.0)^†^	54.1% (±26.3%)	13.6 (±8.3)	5.9 (±3.3)^†^	56.6% (±23.8%)^b^	16.9 (±8.7)	9.9 (±5.6)^†^	41.4% (±20.9%)^c^	0.004^*^
HAM-D	14.5 (±8.1)	8.2 (±5.0)^†^	43.4% (±22.6%)	13.8 (±8.3)	7.5 (±3.8)^†^	45.7% (±24.9%)	14.0 (±8.1)	7.4 (±3.8)^†^	47.1% (±24.4%)^b^	15.7 (±8.0)	11.3 (±6.8)^†^	28.0% (±18.9%)^c^	<0.001^*^
Quality of life													
PDQ-39	58.5 (±17.1)	30.5 (±11.6)^†^	47.9% (±17.8%)	49.7 (±16.2)	27.3 (±10.1)^†^	45.1% (±19.8%)	56.4 (±17.3)	29.1 (±9.7)^†^	48.4% (±18.2%)^b^	64.5 (±17.2)	38.2 (±14.2)^†^	40.8% (±16.3%)^c^	0.002^*^
**Secondary outcome**													
Motor measure													
MDS-UPDRS-II (daily living)	20.3 (±6.2)	11.5 (±5.3)^†^	43.3% (±15.5%)	18.4 (±6.1)	10.3 (±5.0)^†^	44.0% (±15.9%)	19.8 (±6.0)	10.9 (±4.9)^†^	44.9% (±15.2%)^b^	21.6 (±6.4)	12.9 (±6.4)^†^	40.3% (±15.6%)	0.003^*^
MDS-UPDRS-III (on-medicine, motor)	25.8 (±6.8)	16.5 (±8.7)^†^	36.0% (±11.3%)	22.3 (±8.1)	14.6 (±8.3)^†^	34.5% (±13.8%)	24.6 (±6.4)	15.7 (±7.5)^†^	36.2% (±10.3%)	28.5 (±7.2)	18.2 (±9.1)^†^	36.1% (±10.8%)	0.45
MDS-UPDRS-IV (complications)	7.3 (±3.6)	5.1 (±2.3)^†^	30.1% (±10.6%)	5.5 (±2.3)	4.1 (±2.3)^†^	25.5% (±13.1%)	6.9 (±3.0)	5.0 (±2.1)^†^	27.5% (±9.1%)	8.0 (±4.2)	6.1 (±2.7)^†^	23.8% (±11.3%)	0.34
Levodopa-equivalent daily dose, mg	776.5 (±349.9)	315.0 (±170.4)^†^	59.4% (±18.8%)	681.7 (±269.8)	300.6 (±102.9)^†^	55.9% (±15.4%)^a^	738.7 (±312.2)	292.7 (±165.9)^†^	60.4% (±21.3%)^b^	861.9 (±385.6)	421.8 (±185.0)^†^	51.1% (±18.0%)	<0.001^*^
Patient motor diary													
Off time, h/d	5.8 (±1.7)	4.0 (±1.8)^†^	31.0% (±14.8%)	5.1 (±1.6)	3.9 (±1.6)^†^	23.5% (±12.9%)^a^	5.6 (±1.7)	3.8 (±1.6)^†^	32.1% (±15.4%)	6.8 (±1.9)	4.9 (±2.1)^†^	27.9% (±14.9%)	<0.001^*^
On time with troublesome dyskinesia, h/d	4.0 (±1.9)	2.8 (±1.5)^†^	30.0% (±14.9%)	3.5 (±1.9)	2.5 (±1.5)^†^	28.6% (±16.3%)	3.9 (±1.8)	2.8 (±1.3)^†^	28.2% (±14.0%)	4.3 (±2.1)	3.0 (±1.5)^†^	30.2% (±15.1%)	0.71
Neuropsychological evaluation													
MDS-UPDRS-I (non-motor experiences)	14.3 (±4.6)	12.9 (±4.0)^†^	9.8% (±5.1%)	13.4 (±5.3)	11.4 (±4.4)^†^	14.9% (±6.0%)	14.3 (±3.9)	12.5 (±3.5)^†^	12.6% (±4.6%)	16.0 (±5.1)	14.1 (±4.3)	11.9% (±5.1%)	0.052
MMSE	26.4 (±4.9)	27.1 (±3.2)	2.7% (±0.4%)	27.2 (±4.8)	27.9 (±2.5)	2.6% (±0.2%)	26.6 (±4.9)	27.2 (±3.1)	2.3% (±0.4%)	25.3 (±5.8)	26.1 (±3.7)	3.2% (±0.5%)	0.63
MoCA	25.8 (±4.7)	26.0 (±5.8)	0.8% (±0.2%)	26.5 (±4.7)	26.4 (±5.9)	−0.4% (±0.1%)	25.9 (±4.7)	26.1 (±5.8)	0.8% (±0.2%)	24.8 (±4.5)	25.1 (±5.3)	1.2% (±0.3%)	0.79

Refer to S3 Table for detailed within-group comparisons, and S4 Table for detailed between-group comparisons. PD, Parkinson’s disease; STN-DBS, subthalamic nucleus deep brain stimulation; SD, standard deviation; % Improv, improvement (relative change) from baseline; MDS-UPDRS, the Movement Disorder Society-sponsored revision of the Unified Parkinson’s Disease Rating Scale (scale part I, II, III, IV); HAM-A, Hamilton Anxiety Rating Scale; HAM-D, Hamilton Depression Rating Scale; PDQ-39, Parkinson Disease Questionnaire-39; MMSE, Mini-Mental Status Examination; MoCA, Montreal Cognitive Assessment.

† *P* < 0.05 (paired *t t*est; tests for within-group changes in scores from baseline).

^a^*P* < 0.05 (multiple comparisons adjusted by Bonferroni method; tests for pairwise comparison in improvement of short and mid PD duration groups).

^b^*P* < 0.05 (multiple comparisons adjusted by Bonferroni method; tests for pairwise comparison in improvement of mid and long PD duration groups).

* *P* < 0.01 (one-way ANOVA; tests for between-group difference in improvement).

^c^*P* < 0.05 (multiple comparisons adjusted by Bonferroni method; tests for pairwise comparison in improvement of short and long PD duration groups).

### Secondary outcomes and adverse events

**Fig 4:**
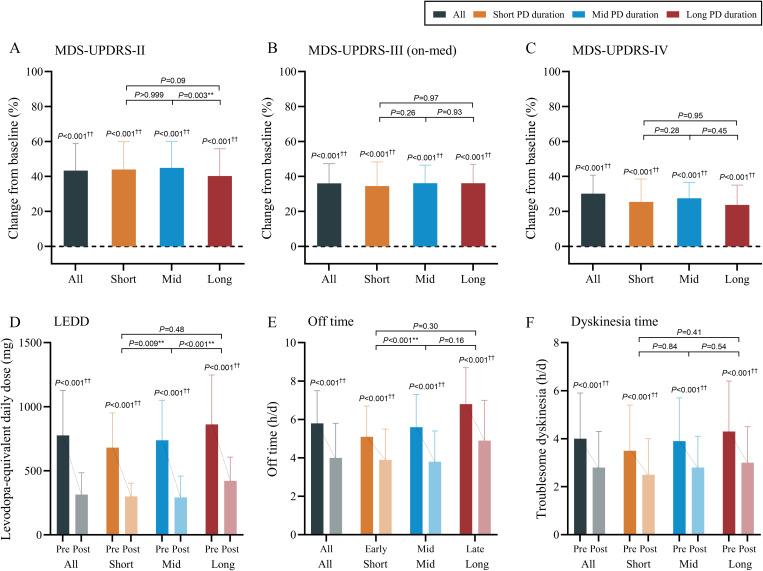
Secondary motor outcomes at 24-month follow-up after the surgery. **A**–**C**: improvements in MDS-UPDRS-III (on-medicine), MDS-UPDRS-II, and MDS-UPDRS-IV; **D**–**F**: changes in LEDD, daily Off time, and daily On time with troublesome dyskinesia. ^†^*P* < 0.05 (within-group comparisons, paired *t t*est); ^††^*P* < 0.01 (within-group comparisons, paired *t* tes*t*); *P < 0.05 (between-group comparisons, one-way ANOVA with multiple comparisons adjusted by Bonferroni method); **P < 0.01 (between-group comparisons, one-way ANOVA with multiple comparisons adjusted by Bonferroni method). The error bars represent the standard deviation (SD). MDS-UPDRS, Movement Disorder Society-sponsored revision of the Unified Parkinson’s Disease Rating Scale (with parts I, II, III, IV); LEDD, levodopa-equivalent daily dose; Pre, Preoperative; Post, Postoperative; Off time, the daily duration of clinically defined “off-medicine” states (h/d); Dyskinesia time, the daily duration of clinically defined “on-medicine” time with troublesome dyskinesia status (h/d).

Secondary motor outcomes (Fig 4) measured by the MDS-UPDRS-II (improved 43.3% ± 15.5%, MD 8.8, 95%CI [8.4, 9.2], *P* < 0.001), and MDS-UPDRS-III (on-medicine; improved 36.0% ± 11.3%, MD 9.3, 95%CI [8.7, 9.9], *P* < 0.001), and the MDS-UPDRS-IV (improved 30.1% ± 10.6%, MD 2.2, 95%CI [2.0, 2.4], *P* < 0.001) significantly improved, whereas the postoperative LEDD (decreased 59.4% ± 18.8%, MD 461.5, 95%CI [439.2, 483.8], *P* < 0.001), off-time (decreased 31.0% ± 14.8%, MD 1.8, 95%CI [1.7, 1.9], *P* < 0.001), and dyskinesia-time (decreased 30.0% ± 14.9%, MD 1.2, 95%CI [1.1, 1.3], *P* < 0.001) also decreased significantly for all patients and for the study groups (all *P* < 0.001).

In terms of secondary neuropsychological evaluations (S2 Fig), the MDS-UPDRS-I score significantly improved in all patients (MD 1.4, 95%CI [1.2, 1.6], **P* *< 0.001) and in the short (MD 2.0, 95%CI [1.0, 3.0], **P* *< 0.001) and mid (MD 1.8, 95%CI [1.1, 2.5], **P* *= 0.02) duration groups, whereas changes in the long duration group did not reach significance (MD 1.9, 95%CI [−0.5, 2.8], **P* *= 0.11). Cognitive functions measured by the MMSE and MoCA did not significantly differ across all patients and groups (all *P* > 0.05). A comparison of the relative changes across the different study groups revealed that the mid duration group had significantly greater improvements in MDS-UPDRS-II scores than did the long duration group (MD 4.6%, 95%CI [2.3%, 6.9%], **P* *= 0.003), greater reductions in LEDD than did the short (MD 4.5%, 95%CI [0.8%, 8.2%], *P* = 0.009) and long (MD 9.3%, 95%CI [6.5%, 12.1%], *P* < 0.001) duration groups, and greater reductions in the daily off-time score than did the short duration group (MD 8.6%, 95%CI [4.9%, 12.3%], *P* < 0.001).

A total of 340 AEs were reported in 302 (17.6%) patients. All these AEs were not life-threatening and resolved without major sequelae, and no surgery-related mortality or morbidity occurred (S5 Table). The most frequently reported AEs were dystonia (46 events reported in 41 [2.4%] patients, all resolved with reprogramming), illusion (37 events reported in 37 [2.2%] patients, all resolved with reprogramming), and infection (36 events reported in 35 [2.0%] patients; all resolved with dressing changes and antibiotics). The prevalence of AEs was comparable across the 3 study groups.

### Correlation analyses

For the primary outcomes, Spearman correlation analysis suggested that the relative changes in the MDS-UPDRS-III (off-medicine) and HAM-A (*r* = 0.159, *P* < 0.001), MDS-UPDRS-III (off-medicine) and HAM-D (*r* = 0.247, *P* < 0.001), MDS-UPDRS-III (off-medicine) and PDQ-39 (*r* = 0.175, *P* < 0.001), HAM-A and HAM-D (*r* = 0.350, *P* < 0.001), HAM-A and PDQ-39 (*r* = 0.088, *P* = 0.017), and HAM-D and PDQ-39 (*r* = 0.165, *P* < 0.001) scores were positively correlated. In addition, it also revealed widely positive relationships (especially extensive connections of motor and neuropsychological outcomes and quality of life) and a minority of negative correlations (such as LEDD with the MDS-UPDRS-IV, *r* = −0.188, *P* < 0.001) with other outcomes. The correlation analyses are shown in S3 Fig and S6 Table.

### Prognostic factors

**Fig 5:**
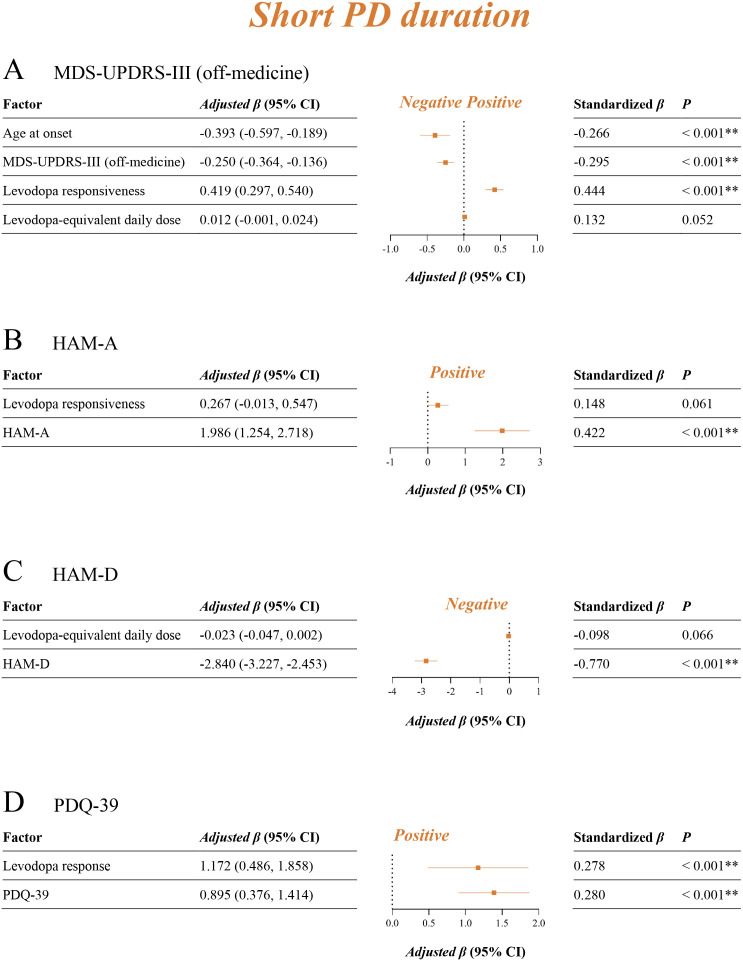
Multivariable linear regression in identifying prognostic factors for the short PD duration group. **A**: Factors influencing improvements in MDS-UPDRS-III (off-medicine); **B**: Factors influencing improvements in HAM-A; **C**: Factors influencing improvements in HAM-D; **D**: Factors influencing improvements in PDQ-39. *P < 0.05 (multivariable linear regression); **P < 0.01 (multivariable linear regression). MDS-UPDRS, Movement Disorder Society-sponsored revision of the Unified Parkinson’s Disease Rating Scale (with parts I, II, III, IV); HAM-A, Hamilton Anxiety Rating Scale; HAM-D, Hamilton Depression Rating Scale; PDQ-39, Parkinson Disease Questionnaire-39.

**Fig 6:**
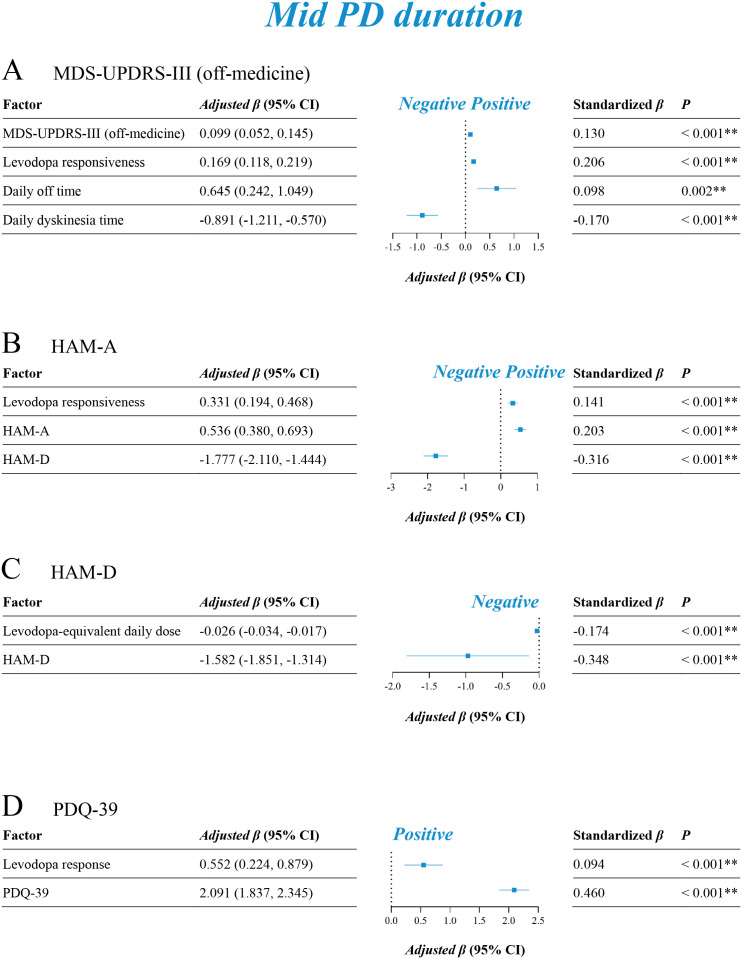
Multivariable linear regression in identifying prognostic factors for the mid PD duration group. **A**: Factors influencing improvements in MDS-UPDRS-III (off-medicine); **B**: Factors influencing improvements in HAM-A; **C**: Factors influencing improvements in HAM-D; **D**: Factors influencing improvements in PDQ-39. *P < 0.05 (multivariable linear regression); **P < 0.01 (multivariable linear regression). MDS-UPDRS, Movement Disorder Society-sponsored revision of the Unified Parkinson’s Disease Rating Scale (with parts I, II, III, IV); HAM-A, Hamilton Anxiety Rating Scale; HAM-D, Hamilton Depression Rating Scale; PDQ-39, Parkinson Disease Questionnaire-39.

**Fig 7:**
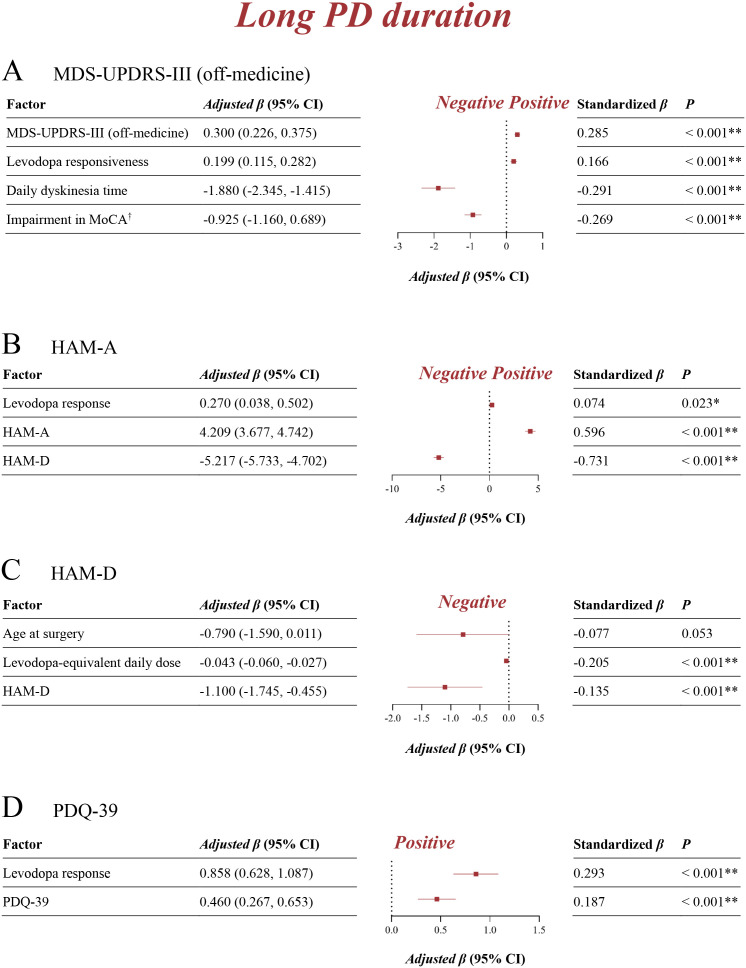
Multivariable linear regression in identifying prognostic factors for the long PD duration group. **A**: Factors influencing improvements in MDS-UPDRS-III (off-medicine); **B**: Factors influencing improvements in HAM-A; **C**: Factors influencing improvements in HAM-D; **D**: Factors influencing improvements in PDQ-39. *P < 0.05 (multivariable linear regression); **P < 0.01 (multivariable linear regression). ^†^ “Impairment in MoCA” is derived by reverse-coding the original MoCA scores (impairments in MoCA = -MoCA), whereas a negative *β* indicates worse motor outcomes with greater cognitive impairment. MDS-UPDRS, Movement Disorder Society-sponsored revision of the Unified Parkinson’s Disease Rating Scale (with parts I, II, III, **IV)**; HAM-A, Hamilton Anxiety Rating Scale; HAM-D, Hamilton Depression Rating Scale; PDQ-39, Parkinson Disease Questionnaire-39.

The univariable linear regression analysis was performed first in identifying potential influential factors of motor outcomes (S7 Table), neuropsychological evaluations (S8 and S9 Tables), and quality of life (S10 Table). Factors with *P* values < 0.10 that might convey important information were then entered into multivariable linear regressions. After adjusting for multivariable models, short (Fig 5), mid (Fig 6), and long (Fig 7) PD duration groups had both consistent and inconsistent prognostic factors. Greater levodopa responses (short: adjusted *β* 0.42, 95% CI [0.30, 0.54], *P* < 0.001; mid: adjusted *β* 0.17, 95% CI [0.12, 0.22], *P* < 0.001; long: adjusted *β* 0.20, 95% CI [0.12, 0.28], *P* < 0.001) were a common positive factor of motor improvement for all three groups. Later age at PD onset (adjusted *β* −0.39, 95% CI [−0.60, −0.20], *P* < 0.001) and higher baseline MDS-UPDRS-III (off-medicine) scores (adjusted *β* −0.25, 95% CI [−0.36, −0.14], *P* < 0.001) were associated with a worse motor response to stimulation in the short duration group. For the mid and long duration groups, higher baseline MDS-UPDRS-III (off-medicine) scores (mid: adjusted *β* 0.10, 95% CI [0.05, 0.15], *P* < 0.001; long: adjusted *β* 0.30, 95% CI [0.23, 0.38], *P* < 0.001) were common positive factors for motor improvement, whereas longer daily dyskinesia time (mid: adjusted *β* −0.89, 95% CI [−1.21, −0.57], *P* < 0.001; long: adjusted *β* −1.88, 95% CI [−2.35, −1.42], *P* < 0.001) was a common negative factor. In addition, longer daily off-time (adjusted *β* 0.65, 95% CI [0.24, 1.05], *P* = 0.002) was positively associated with motor outcomes in the mid duration group, and greater cognitive impairment in MoCA (adjusted *β* −0.93, 95% CI [−1.16, −0.69], *P* < 0.001) were negatively associated with worse motor outcomes in the long duration group.

For neuropsychological evaluations, higher HAM-A scores at baseline (short: adjusted *β* 1.99, 95% CI [1.25, 2.72], *P* < 0.001; mid: adjusted *β* 0.54, 95% CI [0.38, 0.69], *P* < 0.001; long: adjusted *β* 4.21, 95% CI [3.68, 4.74], *P* < 0.001) were associated with greater improvement in anxiety symptoms for all study groups, whereas greater levodopa responses (mid: adjusted *β* 0.33, 95% CI [0.19, 0.47], *P* < 0.001; long: adjusted *β* 0.27, 95% CI [0.04, 0.50], *P* = 0.02) were a positive factor, and higher baseline HAM-D scores (mid: adjusted *β* −1.78, 95% CI [−2.11, −1.44], *P* < 0.001; long: adjusted *β* −5.22, 95% CI [−5.73, −4.70], *P* < 0.001) were a negative factor of anxiety symptoms for the mid and long duration groups. Higher preoperative HAM-D scores (short: adjusted *β* −2.84, 95% CI [−3.23, −2.45], *P* < 0.001; mid: adjusted *β* −1.58, 95% CI [−1.85, −1.31], *P* < 0.001; long: adjusted *β* −1.10, 95% CI [−1.75, −0.46], *P* < 0.001) were a negative factor for depression symptoms in all study groups, and higher LEDD at baseline (mid: adjusted *β* −0.026, 95% CI [−0.034, −0.02], *P* < 0.001; long: adjusted *β* −0.04, 95% CI [−0.06, −0.03], *P* < 0.001) was also a negative prognostic factor for the mid and long duration groups. Furthermore, greater levodopa responses (short: adjusted *β* 1.17, 95% CI [0.49, 1.86], *P* < 0.001; mid: adjusted *β* 0.55, 95% CI [0.22, 0.88], *P* < 0.001; long: adjusted *β* 0.86, 95% CI [0.63, 1.09], *P* < 0.001) and higher baseline PDQ-39 scores (short: adjusted *β* 0.90, 95% CI [0.38, 1.41], *P* < 0.001; mid: adjusted *β* 2.09, 95% CI [1.84, 2.35], *P* < 0.001; long: adjusted *β* 0.46, 95% CI [0.27, 0.65], *P* < 0.001) were associated with greater improvement in quality of life for all study groups.

## Discussion

This large-scale cohort study compared outcomes of DBS for PD at different surgical timings. We revealed that DBS performed after different PD durations (short, mid, or long) could lead to improvements in motor and mood outcomes as well as quality of life while maintaining stable cognition and safety; whereas DBS for patients with mid PD duration (5–10 years) achieved the best outcomes.

Even as a minimally invasive therapy, DBS is usually considered for patients with advanced PD with a long duration in early practice [8,11–16,23]. Previous studies reported significant improvements in STN-DBS for patients with PD, with overall relative changes of 30%−60% in motor symptoms and 20%−50% in quality of life [2,9,10,13–16,55,57,58], and a meta-analysis by Lachenmayer and colleagues estimated a 50.5% reduction in the UPDRS-III score and an improvement in the PDQ-39 score of 22.2% [58], data similar to long duration group findings in our study. With respect to neuropsychological effects, previous findings have varied. A cohort study revealed that mood improved in 50% of patients with PD and the other 50% declined at 12 months after STN-DBS [55], whereas a recent meta-analysis suggested a mild improvement in anxiety and depression at a long follow-up interval (1–3 years) [59]. Our findings suggested that anxiety and depression symptoms improved, whereas stimulation-related neuropsychological AEs were also reported. In addition, positive correlations between improvements in quality of life and motor and mood symptoms were revealed. This suggests the synergistic benefits of alleviating motor symptoms and improving daily activities and psychosocial functions [6,60,61]. With respect to cognitive outcomes, some studies reported cognitive decline in patients with PD after STN-DBS [55,59], whereas other studies in our cohort suggested stable cognitive performance [2,12,14]. The nonmotor symptoms of PD are complex, and the outcomes of DBS can differ significantly across individual patients [2,6]. These variations are influenced by factors such as specific stimulation sites (motor, associative, and limbic regions) and associated circuits [62,63], patient age (especially in cases of cognitive decline) [55,59], adjustments in medication [64], and genetic factors (such as *GBA* mutations) [65]. This complexity necessitates further personalized analyses and thorough discussions regarding potential risks and benefits for each individual [2,6].

Given the positive effects and low surgical risks, increasing enthusiasm has been raised in advocating DBS for patients with shorter duration of PD [18,22,23,66], a trend that was also observed in this study. The most important rationale for early intervention is that it can enhance quality of life in a timely manner and provide occupational benefits as well as possible cost savings for patients with less medication intake [67,68]. Despite several ongoing and finished trials, experience in early DBS is still insufficient [8,17,19,20]. The trial by Schüpbach and colleagues [17] (mean PD duration as 6.8 years; 20 patients), and their subsequent multicenter EARLYSTIM study (mean duration as 7.5 years; 251 patients) included patients with a short PD duration and early motor complications for STN-DBS [20]. They revealed that DBS was superior to medical therapy with respect to quality of life, activities of daily living, and motor symptoms [17,20]. The present study provides potential evidence that DBS for patients with PD after mid duration (5–10 years) might result in best outcomes, while patients with short (but already meet surgical indications as medicine-resistant motor fluctuations or dyskinesias) and long PD duration could receive less but also significant improvement.

However, it should be noted to a different situation in performing DBS for too early PD stages, even at the situation that patients without medication-resistant motor fluctuations or dyskinesias as surgical indications. The trial by Charles and colleagues [69] (2-year follow-up) and Hacker and colleagues [19] (same cohort, 5 years of follow-up) enrolling 30 patients with PD on medication ≥ 6 months but ≤ 4 years (mean 2.1 years) and without motor fluctuations or dyskinesias. Those authors suggested that although patients receiving STN-DBS required lower levodopa doses, there were no significant differences in motor symptoms or quality of life between STN-DBS and medication alone [19,69]. This approach might introduce several risks, including potential confusion of diagnosis only on the basis of initial symptoms [26], insufficient symptom history for proper surgical stratification [23], and unsatisfactory benefits with possible complications [70], which should be alerted as “too early” for DBS.

On the basis of collective experience, several key prognostic factors and their variations among different study groups have been identified, which can help in patient selection and counseling, contributing to future individual outcome improvement [27,28]. Greater levodopa response was considered a unified positive factor of motor and anxiety outcomes as well as quality of life for nearly all groups, a crucial factor also reported in previous studies [27,55,58,71]. A meta-analysis by Lachenmayer and colleagues suggested that levodopa responsiveness was highly predictive of STN-DBS motor outcome across their included studies [58], and their cohort study revealed that a greater levodopa response also predicted improvement in quality of life [58]. With these findings, the levodopa test has been recommended as one of the most important inclusion criteria for DBS, with a minimum of 30%−35% [6,8,72]. This study suggested that its predictive value could be further expanded to include early interventions. However, it should be noted that levodopa responsiveness might not predict long-term outcomes (>3 years) well, which deserves further study [9,27,71]. In addition, longer daily off-time was a positive factor of motor outcomes, whereas longer daily dyskinesia time was negatively associated with motor outcomes. For important dyskinesia, DBS for the globus pallidus internus (GPi) might provide a probable advantage in direct dyskinesia suppression, which could be considered [58,73,74]. With respect to nonmotor outcomes, higher baseline scores on the HAM-A and PDQ-39 correlated with greater improvement, findings that are consistent with those of previous studies [28,55]. However, higher baseline HAM-D scores with higher LEDDs were associated with worse depression outcomes, especially in late surgeries for older patients. Considering the antidepressant effects of dopamine agonists, patients may worsen their depression symptoms with medication reduction, which can more likely occur in patients with large amounts of preoperative medications [6,49,75]. This should be considered in postoperative medication adjustments.

Apart from consistent factors, some prognostic factors could be different or completely opposite for DBS performed at different disease durations. In the mid and long PD duration groups, higher baseline scores on the MDS-UPDRS-III (off-medicine) were associated with better motor response, which was also reported as a positive predictor in previous studies [27,66]. However, our study revealed that higher baseline MDS-UPDRS-III (off-medicine) scores and later disease onset were negative factors in the short PD duration group. For patients with short PD durations but rapidly progressive motor symptoms, Parkinson‒Plus syndrome (also known as atypical Parkinsonism, such as multiple system atrophy, progressive supranuclear palsy, and others) should be considered, as it cannot be effectively alleviated with DBS [26,76]. Thus, unusually rapidly progressing levodopa-resistant symptoms should be considered red flags in surgical patient selection or at least delay the surgery given the difficulty in making a diagnosis on the basis of only early atypical symptoms [11,76]. This may also partially explain why some patients with short PD duration experienced suboptimal outcomes after DBS, attributable to potential alternative diagnosis, and misclassification [26,76]. In addition, increasing cognitive impairment in MoCA was considered related to worse motor symptoms in the long PD duration group. Previous studies have also suggested that cognitive decline is correlated with worse motor outcomes assessed by the MoCA, MMSE, frontal score, and brain imaging findings (e.g., white matter ischemic lesions) [27,77,78], which might be related to combined pathologies involving subcortical nondopaminergic pathways [27]. These findings suggest that preoperative cognitive assessments are important variables in patient selection, especially for older patients with long disease durations. In summary, the current findings highlight the importance of comprehensive assessments before surgery, and it is necessary to separately interpret the prognostic factors of DBS for patients with different disease durations. This point deserves even greater attention for patients undergoing early (PD duration < 5 years) interventions.

This study has several limitations. First, the nonrandomized observational nature of this study introduced selection bias and imbalanced factors for comparisons. We applied relative changes for comparison to increase compatibility and reduce the influence of the baseline differences. Second, loss of follow-up and missing data were inevitable. We performed additional analyses and revealed no significant influence. Third, limited by the data, precious locations of contacts were not available for analysis, which might also involve clues for prognosis [27], and postoperative assessments under nonstimulation conditions were not assessed in routine practice. Although detailed scores in items of MDS-UPDRS were not available for quantitative classification of phenotypes (e.g., tremor dominant and others), we screened clinical manifestations of the included patients from the medical records and revealed comparable breakdown of the symptoms among the three study groups. Fourth, this study included only 2-year outcomes, and future long-term studies are warranted. Notably, longer follow-up studies (e.g., ≥5 years) in patients with short PD duration are essential as a future research direction to confirm diagnostic accuracy and establish long-term prognostic validity. Fifth, although the multicenter design enriched collaborative experience and sample size, a potential imbalance in surgical experience might exist. Thus, we recruited representative experienced DBS centers and analyzed centers and DBS manufactures as potential factors and found no significant differences in outcomes. In addition, the current surgical timing classification is only a given stratification that typically reflects clinical practice; the optimal surgical timing still requires a large amount of experience, and other prognostic factors should also be considered. Finally, a control group with medication only would better compare surgical effects, which requires future evidence from score-matched RCTs.

DBS significantly improved motor, neuropsychological, and quality-of-life outcomes across all PD durations, with the most substantial benefits observed in mid-duration (5–10 years) patients. While levodopa response was a consistent positive prognostic factor for motor response, caution is warranted for short-duration patients with rapidly progressive motor symptoms, as they exhibited less favorable outcomes. Prognostic factors showed variations by surgical timing, emphasizing the importance of individualized patient selection.

## Supporting information

S1 Fig Study centers, locations, and number of patients included at each center. Made with Natural Earth. Free vector and raster map data @ naturalearthdata.com.
(TIF)

S2 Fig Secondary neuropsychological evaluations at 24-month follow-up after the surgery. **A**: changes in MDS-UPDRS-I; **B**: changes in MMSE; **C**: changes in MoCA. ^†^*P* < 0.05 (within-group comparisons, paired *t* test); ^††^*P* < 0.01 (within-group comparisons, paired *t* test). The error bars represent the standard deviation (SD). MDS-UPDRS, Movement Disorder Society-sponsored revision of the Unified Parkinson’s Disease Rating Scale (with parts I, II, III, IV); MMSE, Mini-Mental Status Examination; MoCA, Montreal Cognitive Assessment.
(TIF)

S3 Fig Correlations regarding relative changes in the outcomes. *P < 0.05 (Pearson’s correlation coefficient); **P < 0.01 (Pearson’s correlation coefficient). Refer to S6 Table for the detailed *P* and *r* values.
(TIF)

S1 Table Study centers, locations, region, and number of collected and included patients with Parkinson’s disease (PD) who underwent bilaterial subthalamic nucleus deep brain stimulation (STN-DBS) during 2011−2020 for the Deep Brain Stimulation for Parkinson’s Disease Chinese Collaboration-2 (DBS-PDCC2) study. (DOCX)

S2 Table Non-eligible reasons of excluded samples (*n* = 142) and available information of samples with missing data (*n* = 92). (DOCX)

S3 Table Within-group comparisons of changes in scores for primary and secondary motor, neuropsychological outcomes, and quality of life. (DOCX)

S4 Table Between-group comparisons of improvement (relative change) from baseline (%) for primary and secondary motor, neuropsychological outcomes, and quality of life. (DOCX)

S5 Table Adverse events (AEs) of the included patients with Parkinson’s disease (PD) during the 24 months after bilaterial subthalamic nucleus deep brain stimulation (STN-DBS). (DOCX)

S6 Table Correlation of relative changes in motor, neuropsychological outcomes, and quality of life for the included patients with Parkinson’s disease (PD) at 24 months after bilaterial subthalamic nucleus deep brain stimulation (STN-DBS). (DOCX)

S7 Table Univariable linear regression for potential prognostic factors of motor outcome measured by MDS-UPDRS-III off-medicine relative changes for the included patients with Parkinson’s disease (PD) of different study group at 24 months after subthalamic nucleus deep brain stimulation (STN-DBS). (DOCX)

S8 Table Univariable linear regression for potential prognostic factors of neuropsychological outcomes evaluated by HAM-A relative changes for the included patients with Parkinson’s disease (PD) of different study group at 24 months after subthalamic nucleus deep brain stimulation (STN-DBS). (DOCX)

S9 Table Univariable linear regression for potential prognostic factors of neuropsychological outcomes evaluated by HAM-D relative changes for the included patients with Parkinson’s disease (PD) of different study group at 24 months after subthalamic nucleus deep brain stimulation (STN-DBS). (DOCX)

S10 Table Univariable linear regression for potential prognostic factors of quality of life evaluated by PDQ-39 relative changes for the included patients with Parkinson’s disease (PD) of different study group at 24 months after subthalamic nucleus deep brain stimulation (STN-DBS). (DOCX)

S1 File The prospective analysis plan. (PDF)

S1 Checklist STROBE Statement—checklist of items that should be included in reports of observational studies. (DOCX)

## Abbreviations

AE: adverse event;
HAM-A: Hamilton Anxiety Scale
HAM-D: Hamilton Depression Scale
LEDD: levodopa equivalent daily dose
MDS-UPDRS: Unified Parkinson’s Disease Rating Scale
MMSE: Mini-Mental Status Exam
MoCA: Montreal Cognitive Assessment
PD: Parkinson’s disease
PDQ-39: Parkinson Disease Questionnaire-39
RCT: randomized clinical trials
STN-DBS: subthalamic deep brain stimulation
