# Supplementary figures and images for: Deep brain stimulation surgical timing, outcomes, and prognostic factors in patients with Parkinson’s disease: A Chinese retrospective multicenter cohort study
Source: PLoS Med. 2025 Aug 1;22(8):e1004670. doi: 10.1371/journal.pmed.1004670 (PMC12342336; doi:10.1371/journal.pmed.1004670)

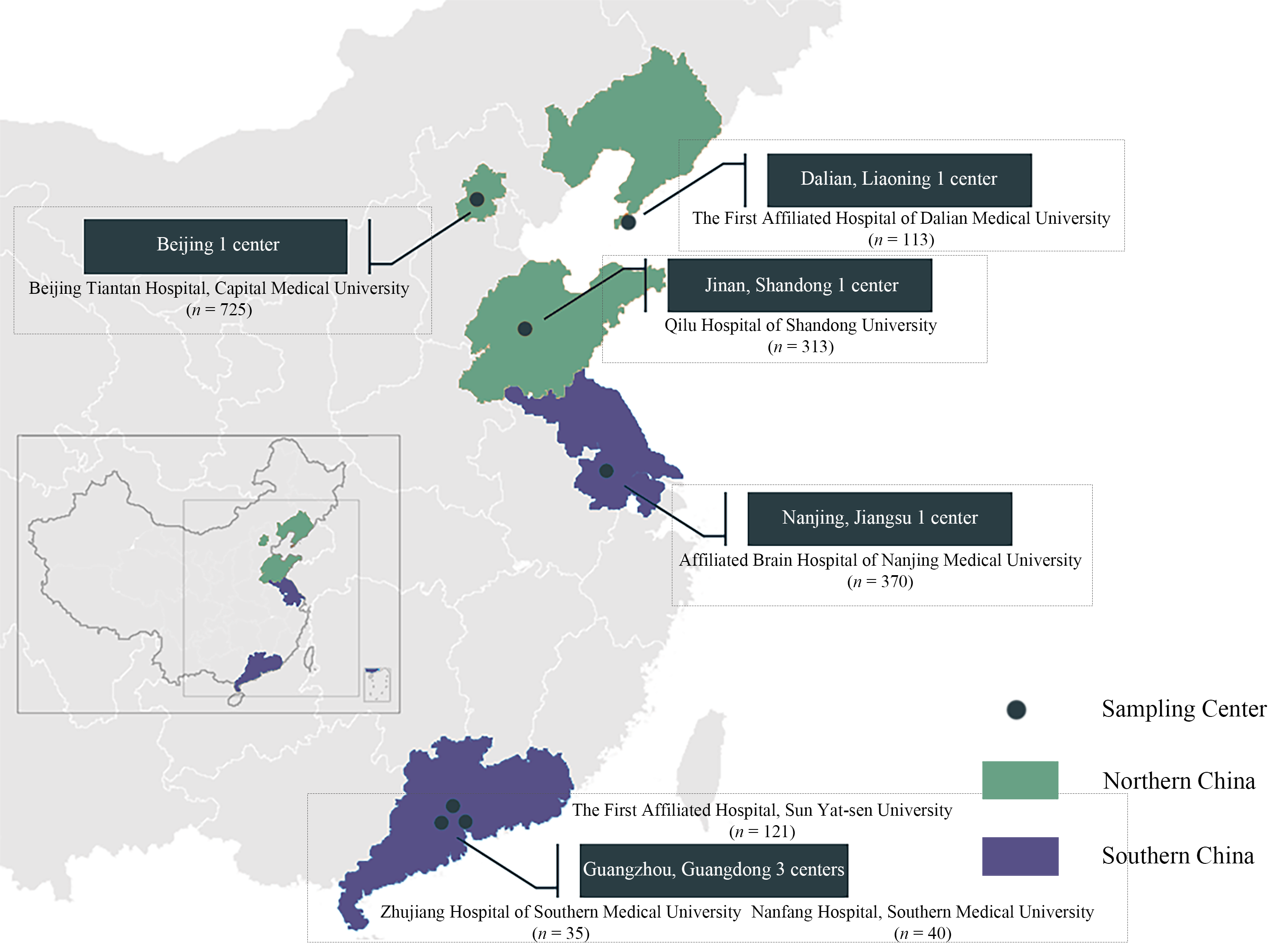

Supplement: S1 Fig — Made with Natural Earth. Free vector and raster map data @ naturalearthdata.com. (TIF) [file pmed.1004670.s001.tif]

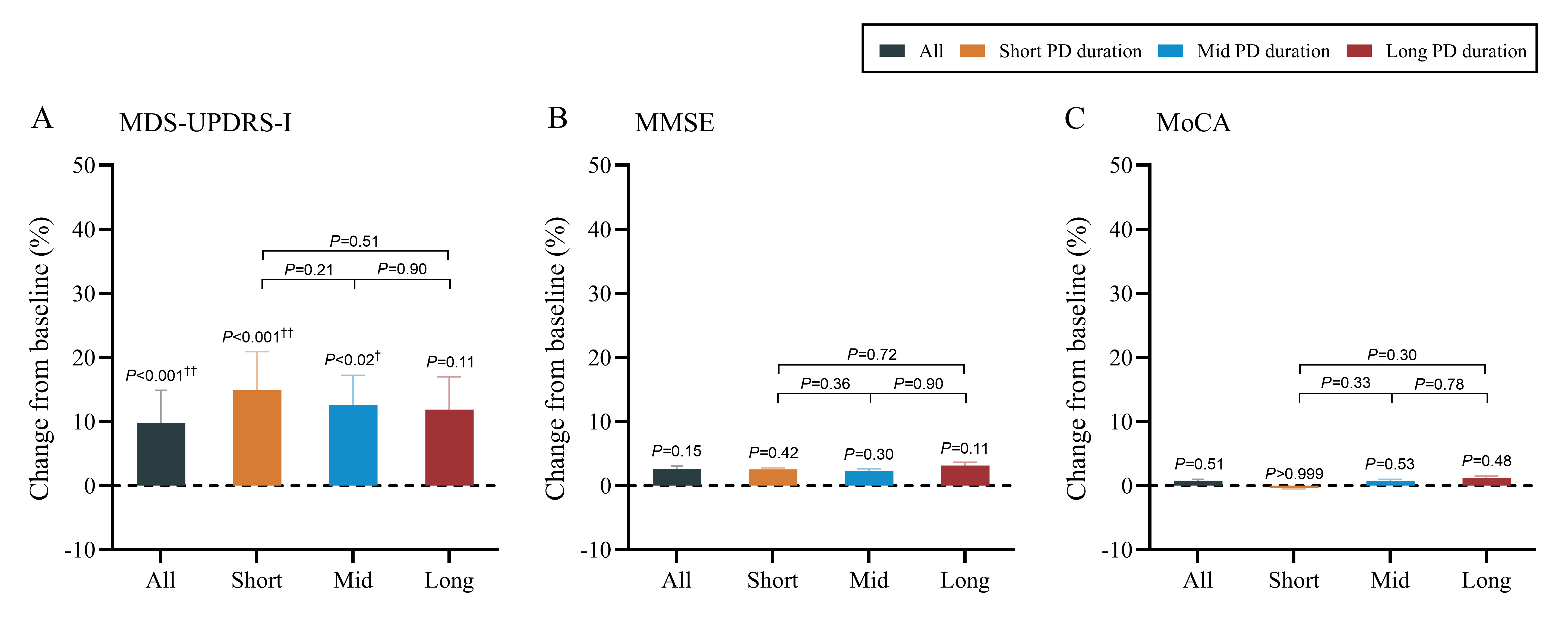

Supplement: S2 Fig — A: changes in MDS-UPDRS-I; B: changes in MMSE; C: changes in MoCA. †P < 0.05 (within-group comparisons, paired t test); ††P < 0.01 (within-group comparisons, paired t test). The error bars represent the standard deviation (SD). MDS-UPDRS, Movement Disorder Society-sponsored revision of the Unified Parkinson’s Disease Rating Scale (with parts I, II, III, IV); MMSE, Mini-Mental Status Examination; MoCA, Montreal Cognitive Assessment. (TIF) [file pmed.1004670.s002.tif]

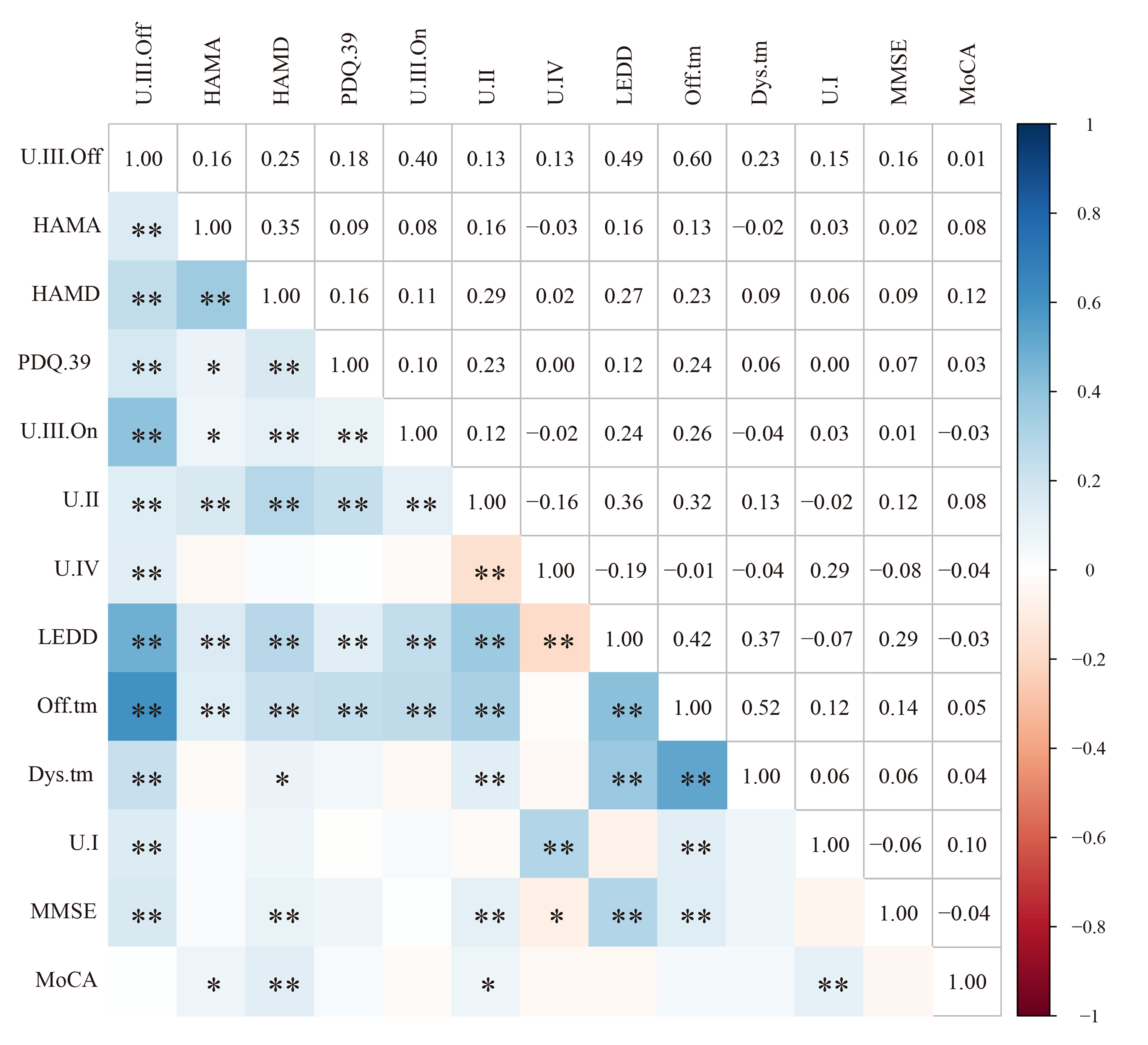

Supplement: S3 Fig — *P < 0.05 (Pearson’s correlation coefficient); **P < 0.01 (Pearson’s correlation coefficient). Refer to S6 Table for the detailed P and r values. (TIF) [file pmed.1004670.s003.tif]
