# Supplementary material for: Deep brain stimulation surgical timing, outcomes, and prognostic factors in patients with Parkinson’s disease: A Chinese retrospective multicenter cohort study
Source: PLoS Med. 2025 Aug 1;22(8):e1004670. doi: 10.1371/journal.pmed.1004670 (PMC12342336; doi:10.1371/journal.pmed.1004670)
Supplement: S1 Table — (DOCX) [file pmed.1004670.s004.docx]

S1 Table. Study centers, locations, region, and number of collected and included patients with Parkinson’s disease (PD) who underwent bilaterial subthalamic nucleus deep brain stimulation (STN-DBS) during 2011-2020 for the Deep Brain Stimulation for Parkinson’s Disease Chinese Collaboration-2 (DBS-PDCC2) study.

|  |  | Number of study patients (*n*) | |
| --- | --- | --- | --- |
| Study center | Location | Enrolled | Included |
| Beijing Tiantan Hospital, Capital Medical University | Beijing (Northern China) | 797 | 725 |
| The First Affiliated Hospital of Dalian Medical University | Dalian, Liaoning (Northern China) | 121 | 113 |
| Qilu Hospital of Shandong University | Jinan, Shandong (Northern China) | 344 | 313 |
| Affiliated Brain Hospital of Nanjing Medical University | Nanjing, Jiangsu (Southern China) | 387 | 370 |
| Zhujiang Hospital of Southern Medical University | Guangzhou, Guangdong (Southern China) | 38 | 35 |
| The First Affiliated Hospital, Sun Yat-sen University | Guangzhou, Guangdong (Southern China) | 130 | 121 |
| Nanfang Hospital, Southern Medical University | Guangzhou, Guangdong (Southern China) | 42 | 40 |
| Total (7 experienced representative centers from wide geographic coverage of northern to southern China | | 1,859 | 1,717 |

PD, Parkinson’s disease; STN-DBS, subthalamic nucleus deep brain stimulation; DBS-PDCC2, Deep Brain Stimulation for Parkinson’s Disease Chinese Collaboration-2
