# Supplementary material for: Deep brain stimulation surgical timing, outcomes, and prognostic factors in patients with Parkinson’s disease: A Chinese retrospective multicenter cohort study
Source: PLoS Med. 2025 Aug 1;22(8):e1004670. doi: 10.1371/journal.pmed.1004670 (PMC12342336; doi:10.1371/journal.pmed.1004670)
Supplement: S2 Table — (DOCX) [file pmed.1004670.s005.docx]

S2 Table. Non-eligible reasons of excluded samples (*n* = 142) and available information of samples with missing data (*n* = 92).

| Non-eligible reason | No. (%) | |
| --- | --- | --- |
| Total excluded | 142 (100.0%; 7.6% of all screened samples) | |
| Without written informed consent | 12 (8.5%) | |
| Underwent DBS in targets other than bilateral STN | 38 (26.8%) | |
| With missing data | 92 (64.8%) | |
| -Missing baseline data | 25 (17.6%) | |
| -Missing follow-up data | 37 (26.1%) | |
| -Less than 2-year follow-up | 30 (21.1%) | |
| Died with no relationship with the DBS | 5 (3.5%) | |
| Could not be contacted due to changed contact information | 21 (14.8%) | |
| Explantation due to infection | 4 (2.8%) | |
| Available information of samples with missing data  (No. of available samples) | No. (%); Mean (±SD); Median (IQR) | *P* (Compare with included samples) |
| Characteristic |  |  |
| Sex (*n* = 92) |  |  |
| Male | 54 (58.7) | 0.66 |
| Female | 38 (41.3) |  |
| Age at surgery, yr (*n* = 92) | 62.5 (55.0, 69.0) | 0.82 |
| Duration of PD, yr (*n* = 89) | 7.0 (5.5, 9.0) | 0.78 |
| Age at PD onset, yr (*n* = 89) | 55.0 (49.0, 60.0) | > 0.999 |
| Young onset PD (*n* = 89) |  |  |
| Yes | 8 (9.0%) | 0.53 |
| No | 81 (91.0%) |  |
| Motor fluctuations (*n* = 92) |  |  |
| Yes | 92 (100.0%) | NA |
| Dyskinesia (*n* = 89) |  |  |
| Yes | 53 (61.2%) | 0.76 |
| No | 36 (38.8%) |  |
| Hoehn & Yahr stage (off-medication) (*n* = 83) | 3.0 (±0.4) | > 0.999 |
| Assessment |  |  |
| Motor measure |  |  |
| MDS-UPDRS-III (off-medicine, motor examination) |  |  |
| Baseline (*n* = 86) | 52.3 (±6.9) | 0.79 |
| At last follow (*n* = 54) | 31.6 (±8.7) | 0.63 |
| MDS-UPDRS-III (on-medicine, motor examination) |  |  |
| Baseline (*n* = 86) | 26.0 (±5.2) | 0.81 |
| At last follow (*n* = 52) | 14.9 (±7.9) | 0.73 |
| Levodopa responsiveness, % |  |  |
| Baseline (*n* = 73) | 50.3 (±6.9) | 0.62 |
| MDS-UPDRS-II (motor experiences of daily living) |  |  |
| Baseline (*n* = 80) | 21.8 (±4.5) | 0.55 |
| At last follow (*n* = 42) | 12.4 (±6.6) | 0.67 |
| MDS-UPDRS-IV (motor complications) |  |  |
| Baseline (*n* = 80) | 6.9 (±4.0) | 0.74 |
| At last follow (*n* = 42) | 5.0 (±1.6) | 0.95 |
| Levodopa-equivalent daily dose, mg |  |  |
| Baseline (*n* = 70) | 828.4 (±220.8) | 0.31 |
| At last follow (*n* = 41) | 293.8 (±82.6) | 0.37 |
| Patient motor diary |  |  |
| Off time, h/d |  |  |
| Baseline (*n* = 76) | 6.0 (±1.9) | 0.68 |
| At last follow (*n* = 44) | 4.1 (±1.7) | > 0.999 |
| On time with troublesome dyskinesia, h/d |  |  |
| Baseline (*n* = 79) | 3.8 (±2.1) | 0.83 |
| At last follow (*n* = 49) | 2.8 (±1.2) | > 0.999 |
| Neuropsychological evaluation |  |  |
| HAM-A |  |  |
| Baseline (*n* = 82) | 16.8 (±2.5) | 0.25 |
| At last follow (*n* = 49) | 8.3 (±5.8) | 0.14 |
| HAM-D |  |  |
| Baseline (*n* = 82) | 15.9 (±3.4) | 0.27 |
| At last follow (*n* = 49) | 8.2 (±5.0) | 0.69 |
| MDS-UPDRS-I (non-motor experiences of daily living) |  |  |
| Baseline (*n* = 76) | 15.0 (±5.2) | 0.66 |
| At last follow (*n* = 44) | 11.3 (±5.9) | 0.84 |
| MMSE |  |  |
| Baseline (*n* = 73) | 27.1 (±4.6) | 0.79 |
| At last follow (*n* = 39) | 27.0 (±2.9) | 0.98 |
| MoCA |  |  |
| Baseline (*n* = 69) | 26.8 (±4.5) | 0.63 |
| At last follow (*n* = 35) | 25.9 (±6.1) | 0.97 |
| Quality of life |  |  |
| PDQ-39 |  |  |
| Baseline (*n* = 85) | 59.0 (±4.8) | 0.56 |
| At last follow (*n* = 53) | 28.1 (±6.2) | 0.62 |

STN, subthalamic nucleus; DBS, deep brain stimulation; SD, standard deviation; IQR, interquartile range (Q1-Q3); MDS-UPDRS, the Movement Disorder Society-sponsored revision of the Unified Parkinson’s Disease Rating Scale (scale part I, II, III, IV); HAM-A, Hamilton Anxiety Rating Scale; HAM-D, Hamilton Depression Rating Scale; MMSE, Mini-Mental Status Examination; MoCA, Montreal Cognitive Assessment; PDQ-39, Parkinson Disease Questionnaire-39.
