# Supplementary material for: Deep brain stimulation surgical timing, outcomes, and prognostic factors in patients with Parkinson’s disease: A Chinese retrospective multicenter cohort study
Source: PLoS Med. 2025 Aug 1;22(8):e1004670. doi: 10.1371/journal.pmed.1004670 (PMC12342336; doi:10.1371/journal.pmed.1004670)
Supplement: S3 Table — (DOCX) [file pmed.1004670.s006.docx]

S3 Table. Within-group comparisons of changes in scores for primary and secondary motor, neuropsychological outcomes, and quality of life.

|  | Mean difference (MD, [95% confidence intervals, CI]) in scores from baseline to 24 months | | | | | | | |
| --- | --- | --- | --- | --- | --- | --- | --- | --- |
| Outcome | Total (*n* = 1,717) | | Short PD duration (*n* = 141) | | Mid PD duration (*n* = 978) | | Long PD duration (*n* = 598) | |
|  | MD (95% CI) | *P* | MD (95% CI) | *P* | MD (95% CI) | *P* | MD (95% CI) | *P* |
| Primary outcome |  |  |  |  |  |  |  |  |
| Motor measure |  |  |  |  |  |  |  |  |
| MDS-UPDRS-III (off-medicine, motor) | 25.1 (24.5, 25.7) | < 0.001** | 20.4 (17.9, 22.9) | < 0.001** | 26.4 (25.5, 27.3) | < 0.001** | 23.7 (22.5, 24.9) | < 0.001** |
| Neuropsychological evaluation |  |  |  |  |  |  |  |  |
| HAM-A | 8.0 (7.5, 8.5) | < 0.001** | 7.2 (5.6, 8.8) | < 0.001** | 7.7 (7.1, 8.3) | < 0.001** | 7.0 (6.2, 7.8) | < 0.001** |
| HAM-D | 6.3 (5.8, 6.8) | < 0.001** | 6.3 (4.8, 7.8) | < 0.001** | 6.6 (6.0, 7.2) | < 0.001** | 4.4 (3.7, 5.1) | < 0.001** |
| Quality of life |  |  |  |  |  |  |  |  |
| PDQ-39 | 28.0 (27.0, 29.0) | < 0.001** | 22.4 (19.5, 25.3) | < 0.001** | 27.3 (26.1, 28.5) | < 0.001** | 26.3 (24.8, 27.8) | < 0.001** |
| Secondary outcome |  |  |  |  |  |  |  |  |
| Motor measure |  |  |  |  |  |  |  |  |
| MDS-UPDRS-II (daily living) | 8.8 (8.4, 9.2) | < 0.001** | 8.1 (6.8, 9.4) | < 0.001** | 8.9 (8.5, 9.3) | < 0.001** | 8.7 (8.0, 9.4) | < 0.001** |
| MDS-UPDRS-III (on-medicine, motor) | 9.3 (8.7, 9.9) | < 0.001** | 7.7 (5.9, 9.5) | < 0.001** | 8.9 (8.3, 9.5) | < 0.001** | 10.3 (9.4, 11.2) | < 0.001** |
| MDS-UPDRS-IV (complications) | 2.2 (2.0, 2.4) | < 0.001** | 1.4 (0.9, 1.9) | < 0.001** | 1.9 (1.7, 2.1) | < 0.001** | 1.9 (1.6, 2.2) | < 0.001** |
| Levodopa-equivalent daily dose, mg | 461.5 (439.2, 483.8) | < 0.001** | 381.1 (335.7, 426.5) | < 0.001** | 446.0 (430.1, 461.9) | < 0.001** | 440.1 (416.3, 463.9) | < 0.001** |
| Patient motor diary |  |  |  |  |  |  |  |  |
| Off time, h/d | 1.8 (1.7, 1.9) | < 0.001** | 1.2 (0.9, 1.5) | < 0.001** | 1.8 (1.7, 1.9) | < 0.001** | 1.9 (1.7, 2.1) | < 0.001** |
| On time with troublesome dyskinesia, h/d | 1.2 (1.1, 1.3) | < 0.001** | 1.0 (0.6, 1.4) | < 0.001** | 1.1 (1.0, 1.2) | < 0.001** | 1.3 (1.1, 1.5) | < 0.001** |
| Neuropsychological evaluation |  |  |  |  |  |  |  |  |
| MDS-UPDRS-I (non-motor experiences) | 1.4 (1.2, 1.6) | < 0.001** | 2.0 (1.0, 3.0) | < 0.001** | 1.8 (1.1, 2.5) | 0.02* | 1.9 (-0.5, 2.8) | 0.11 |
| MMSE | 0.7 (-0.2, 1.6) | 0.15 | 0.7 (-0.5, 1.9) | 0.42 | 0.6 (-0.1, 1.3) | 0.30 | 0.8 (-0.3, 1.6) | 0.11 |
| MoCA | 0.2 (-0.5, 0.9) | 0.51 | -0.1 (-1.0, 0.8) | > 0.999 | 0.2 (-0.4, 0.8) | 0.53 | -0.3 (-0.6, 1.2) | 0.48 |

PD, Parkinson’s disease; STN-DBS, subthalamic nucleus deep brain stimulation; MD, mean difference; CI, confidence interval; MDS-UPDRS, the Movement Disorder Society-sponsored revision of the Unified Parkinson’s Disease Rating Scale (scale part I, II, III, IV); HAM-A, Hamilton Anxiety Rating Scale; HAM-D, Hamilton Depression Rating Scale; PDQ-39, Parkinson Disease Questionnaire-39; MMSE, Mini-Mental Status Examination; MoCA, Montreal Cognitive Assessment. **P* < 0.05 (paired *t*-test; tests for within-group change from baseline); ***P* < 0.01 (paired *t*-test; tests for within-group change from baseline)
