# Supplementary material for: Deep brain stimulation surgical timing, outcomes, and prognostic factors in patients with Parkinson’s disease: A Chinese retrospective multicenter cohort study
Source: PLoS Med. 2025 Aug 1;22(8):e1004670. doi: 10.1371/journal.pmed.1004670 (PMC12342336; doi:10.1371/journal.pmed.1004670)
Supplement: S4 Table — (DOCX) [file pmed.1004670.s007.docx]

S4 Table. Between-group comparisons of improvement (relative change) from baseline (%) for primary and secondary motor, neuropsychological outcomes, and quality of life.

|  | Mean difference (MD, [95% confidence intervals, CI]) in improvement (relative change) from baseline (%) | | | | | |
| --- | --- | --- | --- | --- | --- | --- |
| Outcome | Mid PD duration vs Short PD duration | | Mid PD duration vs Long PD duration | | Short duration vs Long PD duration | |
|  | MD (95% CI) | *P* | MD (95% CI) | *P* | MD (95% CI) | *P* |
| Primary outcome |  |  |  |  |  |  |
| Motor measure |  |  |  |  |  |  |
| MDS-UPDRS-III (off-medicine, motor) | 8.0% (4.7%, 11.3%) | 0.008** | 5.6% (2.8%, 9.4%) | 0.01* | -2.4% (-5.6%, 1.2%) | 0.77 |
| Neuropsychological evaluation |  |  |  |  |  |  |
| HAM-A | 2.5% (-2.1%, 7.1%) | 0.21 | 15.2% (12.3%, 18.1%) | 0.002** | 12.7% (7.6%, 17.8%) | 0.03* |
| HAM-D | 1.4% (-3.5%, 6.3%) | 0.43 | 19.1% (15.6%, 22.6%) | < 0.001** | 17.7% (12.5%, 22.9%) | < 0.001** |
| Quality of life |  |  |  |  |  |  |
| PDQ-39 | 3.3% (-0.2%, 6.8%) | 0.64 | 7.6% (5.2%, 10.0%) | 0.007** | 4.3% (0.5%, 8.1%) | 0.02* |
| Secondary outcome |  |  |  |  |  |  |
| Motor measure |  |  |  |  |  |  |
| MDS-UPDRS-II (daily living) | 0.9% (-2.3%, 4.1%) | > 0.999 | 4.6% (2.3%, 6.9%) | 0.003** | 3.7% (-0.4%, 7.0%) | 0.09 |
| MDS-UPDRS-III (on-medicine, motor) | 1.7% (-1.3%, 4.7%) | 0.26 | 0.1% (-2.1%, 2.3%) | 0.93 | -1.6% (-4.8%, 1.6%) | 0.97 |
| MDS-UPDRS-IV (complications) | 2.0% (-1.6%, 5.6%) | 0.28 | 3.7% (-1.3%, 7.1%) | 0.45 | 1.7% (-1.7%, 5.1%) | 0.95 |
| Levodopa-equivalent daily dose, mg | 4.5% (0.8%, 8.2%) | 0.009** | 9.3% (6.5%, 12.1%) | < 0.001** | 4.8% (-1.6%, 8.0%) | 0.48 |
| Patient motor diary |  |  |  |  |  |  |
| Off time, h/d | 8.6% (4.9%, 12.3%) | < 0.001** | 4.2% (-0.4%, 7.3%) | 0.16 | -4.4% (-8.3%, 0.8%) | 0.30 |
| On time with troublesome dyskinesia, h/d | -0.4% (-4.3%, 3.5%) | 0.84 | -2.0% (-4.9%, 0.9%) | 0.54 | -1.6% (-5.4%, 2.2%) | 0.41 |
| Neuropsychological evaluation |  |  |  |  |  |  |
| MDS-UPDRS-I (non-motor experiences) | -2.3% (-3.7%, 0.2%) | 0.21 | 0.7% (-0.6%, 2.0%) | 0.90 | 3.0% (-1.3%, 4.9%) | 0.51 |
| MMSE | -0.3% (-0.4%, 0.1%) | 0.36 | -0.9% (-1.3%, 0.8%) | 0.90 | -0.6% (-1.2%, 0.4%) | 0.72 |
| MoCA | 1.2% (-0.3%, 2.3%) | 0.33 | -0.4% (-0.7%, 0.3%) | 0.78 | -1.6% (-1.8%, 0.3%) | 0.30 |

PD, Parkinson’s disease; STN-DBS, subthalamic nucleus deep brain stimulation; MD, mean difference; CI, confidence interval; MDS-UPDRS, the Movement Disorder Society-sponsored revision of the Unified Parkinson’s Disease Rating Scale (scale part I, II, III, IV); HAM-A, Hamilton Anxiety Rating Scale; HAM-D, Hamilton Depression Rating Scale; PDQ-39, Parkinson Disease Questionnaire-39; MMSE, Mini-Mental Status Examination; MoCA, Montreal Cognitive Assessment. **P* < 0.05 (one-way ANOVA with multiple comparisons adjusted by Bonferroni method; tests for pairwise between-group comparison in improvement, %); ***P* < 0.01 (one-way ANOVA with multiple comparisons adjusted by Bonferroni method; tests for pairwise between-group comparison in improvement, %)
