# Supplementary material for: Deep brain stimulation surgical timing, outcomes, and prognostic factors in patients with Parkinson’s disease: A Chinese retrospective multicenter cohort study
Source: PLoS Med. 2025 Aug 1;22(8):e1004670. doi: 10.1371/journal.pmed.1004670 (PMC12342336; doi:10.1371/journal.pmed.1004670)
Supplement: S5 Table — (DOCX) [file pmed.1004670.s008.docx]

S5 Table. Adverse events (AEs) of the included patients with Parkinson’s disease (PD) during the 24 months after bilaterial subthalamic nucleus deep brain stimulation (STN-DBS).

|  | No. (% of prevalence) | | | | | | | |  |
| --- | --- | --- | --- | --- | --- | --- | --- | --- | --- |
|  | Total (*n* = 1,717) | | Short PD duration (*n* = 141) | | Mid PD duration (*n* = 978) | | Long PD duration (*n* = 598) | |  |
| Event | No. of patients | No. of events | No. of patients | No. of events | No. of patients | No. of events | No. of patients | No. of events | *P* (for unique patients) |
| Infection | 35 (2.0%) | 36 | 1 (0.7%) | 2 | 18 (1.8%) | 18 | 16 (2.7%) | 16 | 0.31 |
| Cerebral hemorrhage | 11 (0.6%) | 11 | 2 (1.4%) | 2 | 7 (0.7%) | 7 | 2 (0.3%) | 2 | 0.22 |
| Stroke | 7 (0.4%) | 7 | 0 (0%) | 0 | 6 (0.6%) | 6 | 1 (0.2%) | 1 | 0.40 |
| Cerebrospinal fluid leakage | 6 (0.3%) | 6 | 0 (0%) | 0 | 2 (0.2%) | 2 | 4 (0.7%) | 4 | 0.33 |
| Acute pulmonary embolism | 1 (0.1%) | 1 | 0 (0%) | 0 | 0 (0%) | 0 | 1 (0.2%) | 1 | 0.43 |
| Pneumonia | 20 (1.2%) | 20 | 0 (0%) | 0 | 11 (1.1%) | 11 | 9 (1.5%) | 9 | 0.39 |
| Confused state | 16 (0.9%) | 16 | 2 (1.4%) | 2 | 10 (1.0%) | 10 | 4 (0.7%) | 4 | 0.50 |
| Pain | 21 (1.2%) | 28 | 3 (2.1%) | 6 | 10 (1.0%) | 11 | 8 (1.3%) | 11 | 0.38 |
| Dystonia | 41 (2.4%) | 46 | 2 (1.4%) | 4 | 24 (2.5%) | 26 | 15 (2.5%) | 16 | 0.86 |
| Seizure | 15 (0.9%) | 15 | 0 (0%) | 0 | 9 (0.9%) | 9 | 6 (1.0%) | 6 | 0.78 |
| Depression | 33 (1.9%) | 35 | 2 (1.4%) | 2 | 18 (1.8%) | 18 | 13 (2.2%) | 15 | 0.86 |
| Anxiety | 7 (0.4%) | 9 | 0 (0%) | 0 | 3 (0.3%) | 4 | 4 (0.7%) | 5 | 0.58 |
| Impulse control disorder | 12 (0.7%) | 12 | 1 (0.7%) | 1 | 7 (0.7%) | 7 | 4 (0.7%) | 4 | > 0.999 |
| Syncope | 11 (0.6%) | 11 | 3 (2.1%) | 3 | 5 (0.5%) | 5 | 3 (0.5%) | 3 | 0.11 |
| Vasovagal attack | 15 (0.9%) | 15 | 2 (1.4%) | 2 | 10 (1.0%) | 10 | 3 (0.5%) | 3 | 0.35 |
| Cognitive impairment | 4 (0.2%) | 4 | 0 (0%) | 0 | 3 (0.3%) | 3 | 1 (0.2%) | 1 | > 0.999 |
| Illusion | 37 (2.2%) | 37 | 1 (0.7%) | 1 | 19 (1.9%) | 19 | 17 (2.8%) | 17 | 0.27 |
| Manic disorder | 6 (0.3%) | 7 | 2 (1.4%) | 2 | 2 (0.2%) | 2 | 2 (0.3%) | 3 | 0.10 |
| Device related | 24 (1.4%) | 24 | 1 (0.7%) | 1 | 14 (1.4%) | 14 | 9 (1.5%) | 9 | 0.90 |
| Total | 302 (17.6%) | 340 | 22 (15.6%) | 28 | 171 (17.5%) | 182 | 109 (18.2%) | 130 | 0.76 |

AEs, Adverse events; PD, Parkinson’s disease; STN-DBS, subthalamic nucleus deep brain stimulation. Adverse events related either to the surgery, stimulation, or the device were recorded and compared in prevalence for unique patients in the 3 study groups; no significance was revealed (all *P* > 0.05; Pearson’s *χ^2^* or Fisher-Freeman-Halton test, as appropriate).
