# Supplementary material for: Deep brain stimulation surgical timing, outcomes, and prognostic factors in patients with Parkinson’s disease: A Chinese retrospective multicenter cohort study
Source: PLoS Med. 2025 Aug 1;22(8):e1004670. doi: 10.1371/journal.pmed.1004670 (PMC12342336; doi:10.1371/journal.pmed.1004670)
Supplement: S6 Table — (DOCX) [file pmed.1004670.s009.docx]

S6 Table. Correlation of relative changes in motor, neuropsychological outcomes, and quality of life for the included patients with Parkinson’s disease (PD) at 24 months after bilaterial subthalamic nucleus deep brain stimulation (STN-DBS).

|  |  | Correlation | | | | | | | | | | | | |
| --- | --- | --- | --- | --- | --- | --- | --- | --- | --- | --- | --- | --- | --- | --- |
|  |  | U.III.Off | HAMA | HAMD | PDQ.39 | U.III.On | U.II | U.IV | LEDD | Off.tm | Dys.tm | U.I | MMSE | MoCA |
| U.III.Off | *r* | 1 | 0.159** | 0.247** | 0.175** | 0.401** | 0.133** | 0.126** | 0.489** | 0.603** | 0.227** | 0.148** | 0.161** | 0.006 |
|  | *P* | NA | <0.001 | <0.001 | <0.001 | <0.001 | <0.001 | 0.001 | <0.001 | <0.001 | <0.001 | <0.001 | <0.001 | 0.881 |
|  | *N* | 1,717 | 1,717 | 1,717 | 1,717 | 1,717 | 1,717 | 1,717 | 1,717 | 1,717 | 1,717 | 1,717 | 1,717 | 1,717 |
| HAMA | *r* | 0.159** | 1 | 0.350** | 0.088* | 0.075* | 0.163** | -0.033 | 0.155** | 0.133** | -0.024 | 0.029 | 0.024 | 0.079* |
|  | *P* | <0.001 | NA | <0.001 | 0.017 | 0.040 | <0.001 | 0.372 | <0.001 | <0.001 | 0.516 | 0.427 | 0.507 | 0.032 |
|  | *N* | 1,717 | 1,717 | 1,717 | 1,717 | 1,717 | 1,717 | 1,717 | 1,717 | 1,717 | 1,717 | 1,717 | 1,717 | 1,717 |
| HAMD | *r* | 0.247** | 0.350** | 1 | 0.165** | 0.114** | 0.289** | 0.022 | 0.271** | 0.226** | 0.088* | 0.062 | 0.095** | 0.120** |
|  | *P* | <0.001 | <0.001 | NA | <0.001 | 0.002 | <0.001 | 0.549 | <0.001 | <0.001 | 0.016 | 0.089 | 0.010 | 0.001 |
|  | *N* | 1,717 | 1,717 | 1,717 | 1,717 | 1,717 | 1,717 | 1,717 | 1,717 | 1,717 | 1,717 | 1,717 | 1,717 | 1,717 |
| PDQ.39 | *r* | 0.175** | 0.088* | 0.165** | 1 | 0.095** | 0.231** | 0.004 | 0.123** | 0.244** | 0.057 | -0.001 | 0.069 | 0.030 |
|  | *P* | <0.001 | 0.017 | <0.001 | NA | 0.009 | <0.001 | 0.918 | 0.001 | <0.001 | 0.121 | 0.985 | 0.060 | 0.408 |
|  | *N* | 1,717 | 1,717 | 1,717 | 1,717 | 1,717 | 1,717 | 1,717 | 1,717 | 1,717 | 1,717 | 1,717 | 1,717 | 1,717 |
| U.III.On | *r* | 0.401** | 0.075* | 0.114** | 0.095** | 1 | 0.120** | -0.024 | 0.245** | 0.260** | -0.035 | 0.032 | 0.009 | -0.030 |
|  | *P* | <0.001 | 0.040 | 0.002 | 0.009 | NA | 0.001 | 0.515 | <0.001 | <0.001 | 0.337 | 0.383 | 0.802 | 0.422 |
|  | *N* | 1,717 | 1,717 | 1,717 | 1,717 | 1,717 | 1,717 | 1,717 | 1,717 | 1,717 | 1,717 | 1,717 | 1,717 | 1,717 |
| U.II | *r* | 0.133** | 0.163** | 0.289** | 0.231** | 0.120** | 1 | -0.164** | 0.364** | 0.321** | 0.128** | -0.023 | 0.117** | 0.075* |
|  | *P* | <0.001 | <0.001 | <0.001 | <0.001 | 0.001 | NA | <0.001 | <0.001 | <0.001 | <0.001 | 0.538 | 0.001 | 0.040 |
|  | *N* | 1,717 | 1,717 | 1,717 | 1,717 | 1,717 | 1,717 | 1,717 | 1,717 | 1,717 | 1,717 | 1,717 | 1,717 | 1,717 |
| U.IV | *r* | 0.126** | -0.033 | 0.022 | 0.004 | -0.024 | -0.164** | 1 | -0.188** | -0.013 | -0.038 | 0.291** | -0.080* | -0.038 |
|  | *P* | 0.001 | 0.372 | 0.549 | 0.918 | 0.515 | <0.001 | NA | <0.001 | 0.730 | 0.295 | <0.001 | 0.029 | 0.307 |
|  | *N* | 1,717 | 1,717 | 1,717 | 1,717 | 1,717 | 1,717 | 1,717 | 1,717 | 1,717 | 1,717 | 1,717 | 1,717 | 1,717 |
| LEDD | *r* | 0.489** | 0.155** | 0.271** | 0.123** | 0.245** | 0.364** | -0.188** | 1 | 0.417** | 0.371** | -0.069 | 0.291** | -0.031 |
|  | *P* | <0.001 | <0.001 | <0.001 | 0.001 | <0.001 | <0.001 | <0.001 | NA | <0.001 | <0.001 | 0.059 | <0.001 | 0.399 |
|  | *N* | 1,717 | 1,717 | 1,717 | 1,717 | 1,717 | 1,717 | 1,717 | 1,717 | 1,717 | 1,717 | 1,717 | 1,717 | 1,717 |
| Off.tm | *r* | 0.603** | 0.133** | 0.226** | 0.244** | 0.260** | 0.321** | -0.013 | 0.417** | 1 | 0.520** | 0.124** | 0.138** | 0.048 |
|  | *P* | <0.001 | <0.001 | <0.001 | <0.001 | <0.001 | <0.001 | 0.730 | <0.001 | NA | <0.001 | 0.001 | <0.001 | 0.193 |
|  | *N* | 1,717 | 1,717 | 1,717 | 1,717 | 1,717 | 1,717 | 1,717 | 1,717 | 1,717 | 1,717 | 1,717 | 1,717 | 1,717 |
| Dys.tm | *r* | 0.227** | -0.024 | 0.088* | 0.057 | -0.035 | 0.128** | -0.038 | 0.371** | 0.520** | 1 | 0.064 | 0.061 | 0.042 |
|  | *P* | <0.001 | 0.516 | 0.016 | 0.121 | 0.337 | <0.001 | 0.295 | <0.001 | <0.001 | NA | 0.084 | 0.097 | 0.250 |
|  | *N* | 1,717 | 1,717 | 1,717 | 1,717 | 1,717 | 1,717 | 1,717 | 1,717 | 1,717 | 1,717 | 1,717 | 1,717 | 1,717 |
| U.I | *r* | 0.148** | 0.029 | 0.062 | -0.001 | 0.032 | -0.023 | 0.291** | -0.069 | 0.124** | 0.064 | 1 | -0.056 | 0.103** |
|  | *P* | <0.001 | 0.427 | 0.089 | 0.985 | 0.383 | 0.538 | <0.001 | 0.059 | 0.001 | 0.084 | NA | 0.130 | 0.005 |
|  | *N* | 1,717 | 1,717 | 1,717 | 1,717 | 1,717 | 1,717 | 1,717 | 1,717 | 1,717 | 1,717 | 1,717 | 1,717 | 1,717 |
| MMSE | *r* | 0.161** | 0.024 | 0.095** | 0.069 | 0.009 | 0.117** | -0.080* | 0.291** | 0.138** | 0.061 | -0.056 | 1 | -0.043 |
|  | *P* | <0.001 | 0.507 | 0.010 | 0.060 | 0.802 | 0.001 | 0.029 | <0.001 | <0.001 | 0.097 | 0.130 | NA | 0.242 |
|  | *N* | 1,717 | 1,717 | 1,717 | 1,717 | 1,717 | 1,717 | 1,717 | 1,717 | 1,717 | 1,717 | 1,717 | 1,717 | 1,717 |
| MoCA | *r* | 0.006 | 0.079* | 0.120** | 0.030 | -0.030 | 0.075* | -0.038 | -0.031 | 0.048 | 0.042 | 0.103** | -0.043 | 1 |
|  | *P* | 0.881 | 0.032 | 0.001 | 0.408 | 0.422 | 0.040 | 0.307 | 0.399 | 0.193 | 0.250 | 0.005 | 0.242 | NA |
|  | *N* | 1,717 | 1,717 | 1,717 | 1,717 | 1,717 | 1,717 | 1,717 | 1,717 | 1,717 | 1,717 | 1,717 | 1,717 | 1,717 |

PD, Parkinson’s disease; STN-DBS, subthalamic nucleus deep brain stimulation; U., (MDS-UPDRS), the Movement Disorder Society-sponsored revision of the Unified Parkinson’s Disease Rating Scale (scale part I, II, III, IV) assessed at medicine on and off; HAMA, (HAM-A), Hamilton Anxiety Rating Scale; HAMD, (HAM-D), Hamilton Depression Rating Scale; PDQ.39, (PDQ-39), Parkinson Disease Questionnaire-39; LEDD, levodopa-equivalent daily dose, mg; Off.tm, off time, h/d; Dys.tm, on time with troublesome dyskinesia, h/d; MMSE, Mini-Mental Status Examination; MoCA, Montreal Cognitive Assessment. **P* < 0.05 (Pearson’s correlation coefficient); **P* < 0.01 (Pearson’s correlation coefficient).
