# Supplementary material for: Deep brain stimulation surgical timing, outcomes, and prognostic factors in patients with Parkinson’s disease: A Chinese retrospective multicenter cohort study
Source: PLoS Med. 2025 Aug 1;22(8):e1004670. doi: 10.1371/journal.pmed.1004670 (PMC12342336; doi:10.1371/journal.pmed.1004670)
Supplement: S7 Table — (DOCX) [file pmed.1004670.s010.docx]

S7 Table. Univariable linear regression for potential prognostic factors of motor outcome measured by MDS-UPDRS-III off-medicine relative changes for the included patients with Parkinson’s disease (PD) of different study group at 24 months after subthalamic nucleus deep brain stimulation (STN-DBS).

| Group /Variable | *β* (95% CI) | Standardized *β* | *P* |
| --- | --- | --- | --- |
| Short PD duration |  |  |  |
| Sex | -4.293 (-13.703, 5.117) | -0.125 | 0.364 |
| Age at surgery | -0.166 (-0.609, 0.276) | -0.103 | 0.454 |
| Disease duration | -0.704 (-6.592, 5.184) | -0.033 | 0.812 |
| Age at onset | -0.547 (-0.778, -0.316) | -0.369 | < 0.001* |
| Young onset PD | 10.285 (-3.637, 24.206) | 0.199 | 0.144 |
| With dyskinesia | -14.097 (-31.711, 3.516) | -0.281 | 0.113 |
| Hoehn & Yahr stage | 4.256 (-4.462, 12.974) | 0.133 | 0.332 |
| Center of surgery | -1.180 (-3.091, 0.731) | -0.168 | 0.221 |
| DBS manufacture | -2.506 (-8.020, 3.008) | -0.124 | 0.366 |
| MDS-UPDRS-III (off-medicine) | -0.311 (-0.443, -0.179) | -0.367 | < 0.001* |
| MDS-UPDRS-III (on-medicine) | 0.044 (-0.381, 0.470) | 0.029 | 0.835 |
| Levodopa responsiveness | 0.430 (0.289, 0.570) | 0.456 | < 0.001* |
| MDS-UPDRS-II | 0.064 (-0.569, 0.698) | 0.030 | 0.839 |
| MDS-UPDRS-IV | -0.860 (-10.604, 8.884) | -0.088 | 0.836 |
| Levodopa-equivalent daily dose | 0.022 (0.007, 0.037) | 0.245 | 0.003* |
| Daily off time | -1.304 (-4.125, 1.516) | -0.167 | 0.353 |
| Daily dyskinesia time | -1.397 (-3.778, 0.984) | -0.210 | 0.241 |
| HAM-A | -0.212 (-0.930, 0.507) | -0.085 | 0.556 |
| HAM-D | -0.204 (-0.980, 0.572) | -0.076 | 0.600 |
| MDS-UPDRS-I | 0.025 (-0.466, 0.517) | 0.015 | 0.918 |
| Impairment in MMSE^†^ | -0.037 (-0.948, 0.874) | -0.012 | 0.935 |
| Impairment in MoCA^†^ | 0.622 (-0.194, 1.438) | 0.218 | 0.132 |
| PDQ-39 | 0.175 (-0.049, 0.398) | 0.237 | 0.122 |
| Mid PD duration |  |  |  |
| Sex | -1.791 (-4.531, 0.950) | -0.061 | 0.200 |
| Age at surgery | 0.042 (-0.101, 0.185) | 0.027 | 0.568 |
| Disease duration | 0.757 (-0.266, 1.780) | 0.069 | 0.147 |
| Age at onset | 0.027 (-0.116, 0.169) | 0.017 | 0.713 |
| Young onset PD | 2.238 (-2.317, 6.792) | 0.046 | 0.335 |
| With dyskinesia | -3.124 (-7.678, 1.529) | -0.083 | 0.178 |
| Hoehn & Yahr stage | -1.793 (-4.079, 0.492) | -0.073 | 0.124 |
| Center of surgery | 0.182 (-0.477, 0.840) | 0.026 | 0.588 |
| DBS manufacture | 0.975 (-0.656, 2.606) | 0.056 | 0.241 |
| MDS-UPDRS-III (off-medicine) | 0.165 (0.119, 0.211) | 0.218 | < 0.001* |
| MDS-UPDRS-III (on-medicine) | -0.073 (-0.211, 0.065) | -0.049 | 0.302 |
| Levodopa responsiveness | 0.218 (0.169, 0.268) | 0.267 | < 0.001* |
| MDS-UPDRS-II | -0.067 (-0.785, 0.433) | -0.040 | 0.433 |
| MDS-UPDRS-IV | -1.300 (-2.981, 0.380) | -0.188 | 0.127 |
| Levodopa-equivalent daily dose | 0.003 (-0.004, 0.010) | 0.050 | 0.348 |
| Daily off time | 1.093 (0.683, 1.502) | 0.165 | < 0.001* |
| Daily dyskinesia time | -1.220 (-1.540, -0.900) | -0.233 | < 0.001* |
| HAM-A | 0.116 (-0.052, 0.284) | 0.068 | 0.176 |
| HAM-D | 0.148 (-0.057, 0.354) | 0.072 | 0.156 |
| MDS-UPDRS-I | -0.128 (-0.296, 0.039) | -0.076 | 0.132 |
| Impairment in MMSE^†^ | 0.102 (-0.133, 0.338) | 0.043 | 0.394 |
| Impairment in MoCA^†^ | 0.140 (-0.087, 0.367) | 0.061 | 0.227 |
| PDQ-39 | 0.048 (-0.011, 0.107) | 0.086 | 0.112 |
| Long PD duration |  |  |  |
| Sex | 0.289 (-9.378, 9.956) | 0.006 | 0.953 |
| Age at surgery | 0.342 (-0.268, 0.953) | 0.117 | 0.268 |
| Disease duration | 0.111 (-1.404, 1.627) | 0.015 | 0.884 |
| Age at onset | 0.298 (-0.289, 0.885) | 0.106 | 0.315 |
| Young onset PD | -8.678 (-21.770, 4.413) | -0.096 | 0.193 |
| With dyskinesia | -7.351 (-18.078, 3.377) | -0.189 | 0.175 |
| Hoehn & Yahr stage | -3.477 (-7.991, 1.037) | -0.111 | 0.130 |
| Center of surgery | 0.978 (-0.639, 2.594) | 0.087 | 0.234 |
| DBS manufacture | -2.343 (-6.387, 1.700) | -0.084 | 0.254 |
| MDS-UPDRS-III (off-medicine) | 0.356 (0.276, 0.436) | 0.338 | < 0.001* |
| MDS-UPDRS-III (on-medicine) | -0.135 (-0.417, 0.148) | -0.069 | 0.348 |
| Levodopa responsiveness | 0.230 (0.135, 0.324) | 0.191 | < 0.001* |
| MDS-UPDRS-II | -0.088 (-0.583, 0.408) | -0.032 | 0.727 |
| MDS-UPDRS-IV | -0.658 (-1.994, 0.677) | -0.130 | 0.328 |
| Levodopa-equivalent daily dose | -0.005 (-0.017, 0.007) | -0.075 | 0.412 |
| Daily off time | 2.060 (-0.499, 4.619) | 0.219 | 0.112 |
| Daily dyskinesia time | -2.255 (-2.742, -1.769) | -0.349 | < 0.001* |
| HAM-A | 0.186 (-0.250, 0.622) | 0.077 | 0.400 |
| HAM-D | 0.128 (-0.030, 0.286) | 0.065 | 0.113 |
| MDS-UPDRS-I | 0.186 (-0.208, 0.580) | 0.085 | 0.351 |
| Impairment in MMSE^†^ | -0.548 (-1.254, 0.157) | -0.139 | 0.126 |
| Impairment in MoCA^†^ | -0.790 (-1.059, -0.522) | -0.230 | < 0.001* |
| PDQ-39 | 0.101 (-0.097, 0.299) | 0.107 | 0.312 |

PD, Parkinson’s disease; STN-DBS, subthalamic nucleus deep brain stimulation; SD, standard deviation; MDS-UPDRS, the Movement Disorder Society-sponsored revision of the Unified Parkinson’s Disease Rating Scale (scale part I, II, III, IV); HAM-A, Hamilton Anxiety Rating Scale; HAM-D, Hamilton Depression Rating Scale; PDQ-39, Parkinson Disease Questionnaire-39; MMSE, Mini-Mental Status Examination; MoCA, Montreal Cognitive Assessment. * *P* < 0.01 (univariable linear regression). Variables with *P* < 0.10 in the univariable linear regression, which might convey important information, were then entered into the multivariable linear regression (refer to **Figure 3** for final factors included in the multivariable model). ^†^ “Impairment in MMSE/MoCA” is derived by reverse-coding the original MMSE/MoCA scores (impairments in MMSE/MoCA = -MMSE/-MoCA, of which higher values suggested greater cognitive impairment), whereas a negative *β* indicates worse outcomes with greater cognitive impairment.
