# Supplementary material for: Deep brain stimulation surgical timing, outcomes, and prognostic factors in patients with Parkinson’s disease: A Chinese retrospective multicenter cohort study
Source: PLoS Med. 2025 Aug 1;22(8):e1004670. doi: 10.1371/journal.pmed.1004670 (PMC12342336; doi:10.1371/journal.pmed.1004670)
Supplement: S8 Table — (DOCX) [file pmed.1004670.s011.docx]

S8 Table. Univariable linear regression for potential prognostic factors of neuropsychological outcomes evaluated by HAM-A relative changes for the included patients with Parkinson’s disease (PD) of different study group at 24 months after subthalamic nucleus deep brain stimulation (STN-DBS).

| Group /Variable | *β* (95% CI) | Standardized *β* | *P* |
| --- | --- | --- | --- |
| Short PD duration |  |  |  |
| Sex | 5.009 (-12.428, 22.446) | 0.083 | 0.566 |
| Age at surgery | -0.484 (-1.305, 0.338) | -0.168 | 0.242 |
| Disease duration | 7.886 (-4.943, 20.715) | 0.176 | 0.223 |
| Age at onset | -0.538 (-1.346, 0.271) | -0.190 | 0.187 |
| Young onset PD | 6.904 (-19.922, 33.731) | 0.074 | 0.607 |
| With dyskinesia | 33.610 (-10.092, 77.313) | 0.271 | 0.127 |
| Hoehn & Yahr stage | -6.282 (-22.702, 10.137) | -0.110 | 0.446 |
| Center of surgery | -1.652 (-5.105, 1.801) | -0.138 | 0.341 |
| DBS manufacture | -0.613 (-10.722, 9.496) | -0.018 | 0.903 |
| MDS-UPDRS-III (off-medicine) | 0.076 (-0.356, 0.508) | 0.051 | 0.724 |
| MDS-UPDRS-III (on-medicine) | 0.620 (-0.161, 1.401) | 0.224 | 0.117 |
| Levodopa responsiveness | 0.518 (0.229, 0.808) | 0.288 | < 0.001* |
| MDS-UPDRS-II | -0.199 (-1.374, 0.976) | -0.049 | 0.735 |
| MDS-UPDRS-IV | 2.832 (-1.039, 6.703) | 0.208 | 0.148 |
| Levodopa-equivalent daily dose | 0.009 (-0.048, 0.065) | 0.049 | 0.751 |
| Daily off time | -4.592 (-10.686, 1.501) | -0.243 | 0.135 |
| Daily dyskinesia time | 2.132 (-3.256, 7.520) | 0.143 | 0.426 |
| HAM-A | 2.217 (1.520, 2.914) | 0.401 | < 0.001* |
| HAM-D | -0.734 (-2.317, 0.849) | -0.133 | 0.356 |
| MDS-UPDRS-I | 0.642 (-0.318, 1.602) | 0.191 | 0.185 |
| Impairment in MMSE^†^ | -0.997 (-2.734, 0.741) | -0.164 | 0.255 |
| Impairment in MoCA^†^ | -0.280 (-1.752, 1.191) | -0.055 | 0.703 |
| PDQ-39 | 0.122 (-0.236, 0.480) | 0.098 | 0.496 |
| Mid PD duration |  |  |  |
| Sex | 0.380 (-7.139, 7.898) | 0.005 | 0.921 |
| Age at surgery | 0.034 (-0.369, 0.436) | 0.009 | 0.870 |
| Disease duration | 0.904 (-1.801, 3.608) | 0.034 | 0.511 |
| Age at onset | 0.100 (-0.302, 0.502) | 0.025 | 0.625 |
| Young onset PD | -0.379 (-13.415, 12.656) | -0.003 | 0.954 |
| With dyskinesia | 10.346 (-8.550, 29.242) | 0.066 | 0.282 |
| Hoehn & Yahr stage | -3.275 (-9.729, 3.180) | -0.052 | 0.319 |
| Center of surgery | -0.260 (-1.968, 1.448) | -0.015 | 0.765 |
| DBS manufacture | 0.324 (-4.278, 4.926) | 0.007 | 0.890 |
| MDS-UPDRS-III (off-medicine) | 0.191 (-0.049, 0.432) | 0.081 | 0.119 |
| MDS-UPDRS-III (on-medicine) | 0.306 (-0.086, 0.697) | 0.079 | 0.126 |
| Levodopa responsiveness | 0.391 (0.245, 0.536) | 0.166 | < 0.001* |
| MDS-UPDRS-II | 0.375 (-0.115, 0.865) | 0.078 | 0.133 |
| MDS-UPDRS-IV | 2.732 (-10.037, 15.501) | 0.105 | 0.658 |
| Levodopa-equivalent daily dose | -0.008 (-0.030, 0.014) | -0.041 | 0.472 |
| Daily off time | 1.733 (-0.488, 3.953) | 0.084 | 0.126 |
| Daily dyskinesia time | -1.698 (-3.849, 0.453) | -0.094 | 0.121 |
| HAM-A | 0.411 (0.247, 0.575) | 0.156 | < 0.001* |
| HAM-D | -1.604 (-1.943, -1.265) | -0.285 | < 0.001* |
| MDS-UPDRS-I | 0.020 (-0.480, 0.519) | 0.004 | 0.938 |
| Impairment in MMSE^†^ | -0.169 (-1.047, 0.709) | -0.020 | 0.705 |
| Impairment in MoCA^†^ | -0.324 (-1.029, 0.382) | -0.047 | 0.367 |
| PDQ-39 | -0.064 (-0.227, 0.100) | -0.040 | 0.445 |
| Long PD duration |  |  |  |
| Sex | 15.664 (-10.372, 41.700) | 0.118 | 0.235 |
| Age at surgery | -1.000 (-2.690, 0.690) | -0.116 | 0.243 |
| Disease duration | 0.220 (-3.921, 4.361) | 0.011 | 0.916 |
| Age at onset | -0.963 (-2.592, 0.665) | -0.116 | 0.243 |
| Young onset PD | 22.311 (-45.140, 89.762) | 0.065 | 0.513 |
| With dyskinesia | 14.004 (-14.036, 42.044) | 0.136 | 0.321 |
| Hoehn & Yahr stage | 11.130 (-5.614, 27.873) | 0.130 | 0.190 |
| Center of surgery | -2.973 (-8.074, 2.127) | -0.114 | 0.250 |
| DBS manufacture | -10.821 (-27.255, 5.613) | -0.129 | 0.194 |
| MDS-UPDRS-III (off-medicine) | 0.304 (-0.469, 1.076) | 0.077 | 0.437 |
| MDS-UPDRS-III (on-medicine) | -0.158 (-1.195, 0.879) | -0.030 | 0.763 |
| Levodopa responsiveness | 0.874 (0.588, 1.160) | 0.239 | < 0.001* |
| MDS-UPDRS-II | 1.089 (-0.839, 3.018) | 0.111 | 0.265 |
| MDS-UPDRS-IV | -0.124 (-6.535, 6.287) | -0.008 | 0.969 |
| Levodopa-equivalent daily dose | 0.010 (-0.030, 0.050) | 0.050 | 0.628 |
| Daily off time | 0.898 (-8.542, 10.338) | 0.024 | 0.850 |
| Daily dyskinesia time | -0.891 (-5.411, 3.629) | -0.054 | 0.694 |
| HAM-A | 1.569 (1.016, 2.123) | 0.222 | < 0.001* |
| HAM-D | -2.918 (-3.441, -2.394) | -0.409 | < 0.001* |
| MDS-UPDRS-I | 0.365 (-0.968, 1.697) | 0.054 | 0.588 |
| Impairment in MMSE^†^ | -2.382 (-5.513, 0.748) | -0.149 | 0.134 |
| Impairment in MoCA^†^ | -1.732 (-4.210, 0.747) | -0.137 | 0.169 |
| PDQ-39 | 0.170 (-0.389, 0.728) | 0.060 | 0.548 |

PD, Parkinson’s disease; STN-DBS, subthalamic nucleus deep brain stimulation; SD, standard deviation; MDS-UPDRS, the Movement Disorder Society-sponsored revision of the Unified Parkinson’s Disease Rating Scale (scale part I, II, III, IV); HAM-A, Hamilton Anxiety Rating Scale; HAM-D, Hamilton Depression Rating Scale; PDQ-39, Parkinson Disease Questionnaire-39; MMSE, Mini-Mental Status Examination; MoCA, Montreal Cognitive Assessment. **P* < 0.01 (univariable linear regression). Variables with *P* < 0.10 in the univariable linear regression, which might convey important information, were then entered into the multivariable linear regression (refer to **Figure 3** for final factors included in the multivariable model). ^†^ “Impairment in MMSE/MoCA” is derived by reverse-coding the original MMSE/MoCA scores (impairments in MMSE/MoCA = -MMSE/-MoCA, of which higher values suggested greater cognitive impairment), whereas a negative *β* indicates worse outcomes with greater cognitive impairment.
