# Supplementary material for: Deep brain stimulation surgical timing, outcomes, and prognostic factors in patients with Parkinson’s disease: A Chinese retrospective multicenter cohort study
Source: PLoS Med. 2025 Aug 1;22(8):e1004670. doi: 10.1371/journal.pmed.1004670 (PMC12342336; doi:10.1371/journal.pmed.1004670)
Supplement: S9 Table — (DOCX) [file pmed.1004670.s012.docx]

S9 Table. Univariable linear regression for potential prognostic factors of neuropsychological outcomes evaluated by HAM-D relative changes for the included patients with Parkinson’s disease (PD) of different study group at 24 months after subthalamic nucleus deep brain stimulation (STN-DBS).

| Group /Variable | *β* (95% CI) | Standardized *β* | *P* |
| --- | --- | --- | --- |
| Short PD duration |  |  |  |
| Sex | 7.155 (-13.859, 28.170) | 0.098 | 0.497 |
| Age at surgery | -0.462 (-1.458, 0.535) | -0.133 | 0.356 |
| Disease duration | 4.086 (-13.458, 21.629) | 0.067 | 0.642 |
| Age at onset | -0.572 (-1.551, 0.408) | -0.167 | 0.247 |
| Young onset PD | 8.695 (-23.672, 41.062) | 0.078 | 0.592 |
| With dyskinesia | 2.602 (-31.073, 36.277) | 0.028 | 0.876 |
| Hoehn & Yahr stage | 9.662 (-10.078, 29.401) | 0.141 | 0.330 |
| Center of surgery | -1.607 (-6.046, 2.832) | -0.104 | 0.470 |
| DBS manufacture | 4.309 (-7.599, 16.216) | 0.104 | 0.470 |
| MDS-UPDRS-III (off-medicine) | 0.340 (-0.173, 0.853) | 0.189 | 0.189 |
| MDS-UPDRS-III (on-medicine) | 0.475 (-0.540, 1.490) | 0.135 | 0.351 |
| Levodopa responsiveness | 0.431 (-0.150, 1.012) | 0.211 | 0.142 |
| MDS-UPDRS-II | 0.494 (-0.918, 1.906) | 0.101 | 0.485 |
| MDS-UPDRS-IV | -1.736 (-4.031, 0.560) | -0.214 | 0.135 |
| Levodopa-equivalent daily dose | -0.036 (-0.074, 0.003) | -0.153 | 0.069 |
| Daily off time | -3.126 (-9.539, 3.287) | -0.160 | 0.330 |
| Daily dyskinesia time | 3.479 (-1.218, 8.176) | 0.262 | 0.141 |
| HAM-A | -1.514 (-3.468, 0.440) | -0.219 | 0.126 |
| HAM-D | -2.866 (-3.255, -2.477) | -0.777 | < 0.001* |
| MDS-UPDRS-I | 0.660 (-0.448, 1.768) | 0.170 | 0.237 |
| Impairment in MMSE^†^ | -0.896 (-3.006, 1.214) | -0.122 | 0.397 |
| Impairment in MoCA^†^ | -0.708 (-2.475, 1.058) | -0.116 | 0.424 |
| PDQ-39 | 0.274 (-0.152, 0.701) | 0.183 | 0.202 |
| Mid PD duration |  |  |  |
| Sex | -0.756 (-7.347, 5.836) | -0.012 | 0.822 |
| Age at surgery | 0.269 (-0.082, 0.620) | 0.078 | 0.132 |
| Disease duration | -1.122 (-3.643, 1.399) | -0.045 | 0.382 |
| Age at onset | 0.272 (-0.077, 0.621) | 0.079 | 0.126 |
| Young onset PD | -1.719 (-13.174, 9.737) | -0.015 | 0.768 |
| With dyskinesia | 7.431 (-7.252, 22.115) | 0.061 | 0.320 |
| Hoehn & Yahr stage | -4.220 (-9.772, 1.333) | -0.077 | 0.136 |
| Center of surgery | -0.467 (-2.065, 1.131) | -0.030 | 0.566 |
| DBS manufacture | 1.165 (-2.865, 5.195) | 0.029 | 0.570 |
| MDS-UPDRS-III (off-medicine) | 0.128 (-0.079, 0.334) | 0.063 | 0.225 |
| MDS-UPDRS-III (on-medicine) | 0.105 (-0.236, 0.445) | 0.031 | 0.546 |
| Levodopa responsiveness | 0.100 (-0.106, 0.307) | 0.049 | 0.342 |
| MDS-UPDRS-II | 0.295 (-0.138, 0.727) | 0.069 | 0.181 |
| MDS-UPDRS-IV | 0.992 (-8.258, 10.243) | 0.053 | 0.824 |
| Levodopa-equivalent daily dose | -0.040 (-0.049, -0.031) | -0.274 | < 0.001* |
| Daily off time | 0.170 (-1.778, 2.117) | 0.009 | 0.864 |
| Daily dyskinesia time | 1.252 (-0.411, 2.914) | 0.090 | 0.140 |
| HAM-A | -0.358 (-0.836, 0.120) | -0.076 | 0.141 |
| HAM-D | -1.809 (-2.070, -1.547) | -0.398 | < 0.001* |
| MDS-UPDRS-I | -0.078 (-0.516, 0.359) | -0.018 | 0.725 |
| Impairment in MMSE^†^ | -0.212 (-0.983, 0.559) | -0.028 | 0.589 |
| Impairment in MoCA^†^ | -0.343 (-0.962, 0.276) | -0.056 | 0.276 |
| PDQ-39 | 0.035 (-0.109, 0.178) | 0.025 | 0.635 |
| Long PD duration |  |  |  |
| Sex | 16.167 (-14.574, 46.909) | 0.102 | 0.299 |
| Age at surgery | -0.746 (-1.569, 0.077) | -0.073 | 0.076 |
| Disease duration | -3.728 (-8.597, 1.142) | -0.148 | 0.132 |
| Age at onset | -1.228 (-3.133, 0.677) | -0.125 | 0.204 |
| Young onset PD | -2.295 (-74.690, 70.100) | -0.006 | 0.950 |
| With dyskinesia | 28.351 (-27.524, 84.226) | 0.138 | 0.313 |
| Hoehn & Yahr stage | 13.298 (-6.620, 33.216) | 0.129 | 0.188 |
| Center of surgery | -2.170 (-8.410, 4.070) | -0.068 | 0.492 |
| DBS manufacture | -1.745 (-21.339, 17.849) | -0.017 | 0.860 |
| MDS-UPDRS-III (off-medicine) | 0.452 (-0.452, 1.355) | 0.097 | 0.324 |
| MDS-UPDRS-III (on-medicine) | 0.181 (-1.005, 1.367) | 0.030 | 0.763 |
| Levodopa responsiveness | 0.213 (-0.657, 1.082) | 0.048 | 0.628 |
| MDS-UPDRS-II | 1.453 (-0.758, 3.664) | 0.127 | 0.195 |
| MDS-UPDRS-IV | 4.716 (-9.396, 18.827) | 0.131 | 0.499 |
| Levodopa-equivalent daily dose | -0.049 (-0.065, -0.032) | -0.232 | < 0.001* |
| Daily off time | 4.897 (-5.484, 15.279) | 0.116 | 0.350 |
| Daily dyskinesia time | 5.415 (-4.066, 14.896) | 0.155 | 0.257 |
| HAM-A | -1.128 (-2.748, 0.493) | -0.135 | 0.171 |
| HAM-D | -1.329 (-1.975, -0.682) | -0.163 | < 0.001* |
| MDS-UPDRS-I | 0.745 (-0.820, 2.311) | 0.093 | 0.347 |
| Impairment in MMSE^†^ | -0.819 (-4.574, 2.935) | -0.043 | 0.666 |
| Impairment in MoCA^†^ | -1.942 (-4.896, 1.012) | -0.127 | 0.195 |
| PDQ-39 | 0.512 (-0.140, 1.163) | 0.152 | 0.123 |

PD, Parkinson’s disease; STN-DBS, subthalamic nucleus deep brain stimulation; SD, standard deviation; MDS-UPDRS, the Movement Disorder Society-sponsored revision of the Unified Parkinson’s Disease Rating Scale (scale part I, II, III, IV); HAM-A, Hamilton Anxiety Rating Scale; HAM-D, Hamilton Depression Rating Scale; PDQ-39, Parkinson Disease Questionnaire-39; MMSE, Mini-Mental Status Examination; MoCA, Montreal Cognitive Assessment. **P* < 0.01 (univariable linear regression). Variables with *P* < 0.10 in the univariable linear regression, which might convey important information, were then entered into the multivariable linear regression (refer to **Figure 3** for final factors included in the multivariable model). ^†^ “Impairment in MMSE/MoCA” is derived by reverse-coding the original MMSE/MoCA scores (impairments in MMSE/MoCA = -MMSE/-MoCA, of which higher values suggested greater cognitive impairment), whereas a negative *β* indicates worse outcomes with greater cognitive impairment.
