# Supplementary material for: Deep brain stimulation surgical timing, outcomes, and prognostic factors in patients with Parkinson’s disease: A Chinese retrospective multicenter cohort study
Source: PLoS Med. 2025 Aug 1;22(8):e1004670. doi: 10.1371/journal.pmed.1004670 (PMC12342336; doi:10.1371/journal.pmed.1004670)
Supplement: S10 Table — (DOCX) [file pmed.1004670.s013.docx]

S10 Table. Univariable linear regression for potential prognostic factors of quality of life evaluated by PDQ-39 relative changes for the included patients with Parkinson’s disease (PD) of different study group at 24 months after subthalamic nucleus deep brain stimulation (STN-DBS).

| Group /Variable | *β* (95% CI) | Standardized *β* | *P* |
| --- | --- | --- | --- |
| Short PD duration |  |  |  |
| Sex | 3.738 (-44.074, 51.550) | 0.024 | 0.875 |
| Age at surgery | -1.020 (-3.185, 1.145) | -0.143 | 0.347 |
| Disease duration | 14.495 (-16.058, 45.048) | 0.144 | 0.344 |
| Age at onset | -1.065 (-3.198, 1.069) | -0.152 | 0.320 |
| Young onset PD | 40.574 (-28.638, 109.787) | 0.177 | 0.244 |
| With dyskinesia | 27.838 (-103.184, 158.859) | 0.078 | 0.668 |
| Hoehn & Yahr stage | 2.883 (-41.120, 46.886) | 0.020 | 0.896 |
| Center of surgery | -2.545 (-11.615, 6.526) | -0.086 | 0.574 |
| DBS manufacture | -4.708 (-32.210, 22.793) | -0.053 | 0.732 |
| MDS-UPDRS-III (off-medicine) | -0.230 (-1.450, 0.990) | -0.058 | 0.705 |
| MDS-UPDRS-III (on-medicine) | 0.868 (-1.342, 3.078) | 0.120 | 0.433 |
| Levodopa responsiveness | 1.646 (0.994, 2.298) | 0.390 | < 0.001* |
| MDS-UPDRS-II | 1.085 (-2.145, 4.315) | 0.103 | 0.502 |
| MDS-UPDRS-IV | -0.655 (-8.262, 6.952) | -0.049 | 0.856 |
| Levodopa-equivalent daily dose | 0.052 (-0.100, 0.204) | 0.106 | 0.495 |
| Daily off time | -5.911 (-25.130, 13.307) | -0.110 | 0.535 |
| Daily dyskinesia time | -1.946 (-17.688, 13.797) | -0.045 | 0.803 |
| HAM-A | 0.403 (-4.640, 5.446) | 0.025 | 0.873 |
| HAM-D | -2.342 (-6.445, 1.760) | -0.173 | 0.256 |
| MDS-UPDRS-I | 1.502 (-0.901, 3.904) | 0.189 | 0.214 |
| Impairment in MMSE^†^ | 1.564 (-3.722, 6.850) | 0.091 | 0.554 |
| Impairment in MoCA^†^ | 1.214 (-2.820, 5.249) | 0.092 | 0.547 |
| PDQ-39 | 1.251 (0.758, 1.744) | 0.391 | < 0.001* |
| Mid PD duration |  |  |  |
| Sex | 7.360 (-14.181, 28.901) | 0.036 | 0.502 |
| Age at surgery | 0.589 (-0.563, 1.741) | 0.053 | 0.315 |
| Disease duration | 2.587 (-5.707, 10.881) | 0.032 | 0.540 |
| Age at onset | 0.537 (-0.613, 1.687) | 0.049 | 0.359 |
| Young onset PD | 0.805 (-36.261, 37.872) | 0.002 | 0.966 |
| With dyskinesia | -15.055 (-69.575, 39.464) | -0.033 | 0.587 |
| Hoehn & Yahr stage | -2.543 (-20.900, 15.813) | -0.014 | 0.785 |
| Center of surgery | 0.462 (-4.524, 5.448) | 0.010 | 0.855 |
| DBS manufacture | 1.481 (-11.644, 14.607) | 0.012 | 0.824 |
| MDS-UPDRS-III (off-medicine) | 0.074 (-0.592, 0.740) | 0.012 | 0.828 |
| MDS-UPDRS-III (on-medicine) | -0.178 (-1.291, 0.935) | -0.017 | 0.754 |
| Levodopa responsiveness | 1.017 (0.653, 1.380) | 0.173 | < 0.001* |
| MDS-UPDRS-II | 0.837 (-0.558, 2.232) | 0.062 | 0.239 |
| MDS-UPDRS-IV | 0.229 (-6.092, 6.551) | 0.019 | 0.940 |
| Levodopa-equivalent daily dose | 0.040 (-0.024, 0.105) | 0.069 | 0.221 |
| Daily off time | 5.602 (-1.390, 12.593) | 0.089 | 0.116 |
| Daily dyskinesia time | -1.163 (-7.923, 5.598) | -0.021 | 0.735 |
| HAM-A | 0.141 (-1.497, 1.779) | 0.009 | 0.866 |
| HAM-D | 0.628 (-0.992, 2.247) | 0.040 | 0.446 |
| MDS-UPDRS-I | -0.544 (-1.950, 0.862) | -0.040 | 0.447 |
| Impairment in MMSE^†^ | 0.101 (-2.398, 2.599) | 0.004 | 0.937 |
| Impairment in MoCA^†^ | -0.352 (-2.354, 1.649) | -0.018 | 0.729 |
| PDQ-39 | 2.164 (1.913, 2.416) | 0.476 | < 0.001* |
| Long PD duration |  |  |  |
| Sex | 4.630 (-19.138, 28.398) | 0.039 | 0.700 |
| Age at surgery | -0.880 (-2.394, 0.635) | -0.116 | 0.252 |
| Disease duration | -1.023 (-4.729, 2.683) | -0.056 | 0.585 |
| Age at onset | -0.656 (-2.120, 0.807) | -0.090 | 0.375 |
| Young onset PD | 30.577 (-23.245, 84.400) | 0.114 | 0.262 |
| With dyskinesia | -8.407 (-51.724, 34.910) | -0.054 | 0.699 |
| Hoehn & Yahr stage | 2.775 (-12.770, 18.321) | 0.036 | 0.724 |
| Center of surgery | 0.148 (-4.884, 5.181) | 0.006 | 0.953 |
| DBS manufacture | 5.278 (-9.929, 20.485) | 0.070 | 0.493 |
| MDS-UPDRS-III (off-medicine) | 0.528 (-0.173, 1.228) | 0.150 | 0.138 |
| MDS-UPDRS-III (on-medicine) | -0.152 (-1.056, 0.752) | -0.034 | 0.739 |
| Levodopa responsiveness | 1.039 (0.819, 1.259) | 0.355 | < 0.001* |
| MDS-UPDRS-II | -1.182 (-2.879, 0.515) | -0.139 | 0.170 |
| MDS-UPDRS-IV | 2.575 (-3.691, 8.842) | 0.163 | 0.406 |
| Levodopa-equivalent daily dose | -0.005 (-0.038, 0.029) | -0.028 | 0.786 |
| Daily off time | 1.866 (-7.497, 11.229) | 0.051 | 0.692 |
| Daily dyskinesia time | 0.773 (-6.389, 7.935) | 0.030 | 0.829 |
| HAM-A | 0.823 (-0.440, 2.087) | 0.130 | 0.199 |
| HAM-D | 0.731 (-0.622, 2.083) | 0.108 | 0.286 |
| MDS-UPDRS-I | -0.118 (-1.309, 1.073) | -0.020 | 0.844 |
| Impairment in MMSE^†^ | 0.865 (-2.150, 3.880) | 0.058 | 0.570 |
| Impairment in MoCA^†^ | -1.222 (-3.570, 1.126) | -0.104 | 0.304 |
| PDQ-39 | 0.701 (0.510, 0.891) | 0.284 | < 0.001* |

PD, Parkinson’s disease; STN-DBS, subthalamic nucleus deep brain stimulation; SD, standard deviation; MDS-UPDRS, the Movement Disorder Society-sponsored revision of the Unified Parkinson’s Disease Rating Scale (scale part I, II, III, IV); HAM-A, Hamilton Anxiety Rating Scale; HAM-D, Hamilton Depression Rating Scale; PDQ-39, Parkinson Disease Questionnaire-39; MMSE, Mini-Mental Status Examination; MoCA, Montreal Cognitive Assessment. **P* < 0.01 (univariable linear regression). Variables with *P* < 0.10 in the univariable linear regression, which might convey important information, were then entered into the multivariable linear regression (refer to **Figure 3** for final factors included in the multivariable model). ^†^ “Impairment in MMSE/MoCA” is derived by reverse-coding the original MMSE/MoCA scores (impairments in MMSE/MoCA = -MMSE/-MoCA, of which higher values suggested greater cognitive impairment), whereas a negative *β* indicates worse outcomes with greater cognitive impairment.
