# Supplementary material for: Deep brain stimulation surgical timing, outcomes, and prognostic factors in patients with Parkinson’s disease: A Chinese retrospective multicenter cohort study
Source: PLoS Med. 2025 Aug 1;22(8):e1004670. doi: 10.1371/journal.pmed.1004670 (PMC12342336; doi:10.1371/journal.pmed.1004670)
Supplement: S1 File — (PDF) [file pmed.1004670.s014.pdf]

The prospective analysis plan (SAP) of the Deep Brain Stimulation for Parkinson's Disease Chinese Collaboration-2 (DBS-PDCC-2) study.

Statistical analysis plan (SAP) of the Deep Brain Stimulation for Parkinson' s Disease Chinese Collaboration-2 (DBS-PDCC-2) study

SAP Date

March 10th, 2023 <*final changed date*>

SAP Version

V1.2, reviewed & approved <*final version*>

Proposers

Dr. Shu Wang of Department of Neurosurgery, Beijing Tiantan Hospital, Capital Medical University

Reviewers

Prof. Anxin Wang and his team members of China National Clinical Research Center for Neurological Diseases, Beijing Tiantan Hospital, Capital Medical University; Prof. Yali Liu and her team members of the Center for Clinical Epidemiology and Evidence-based Medicine, Beijing Children's Hospital, Capital Medical University

Details

The assumption of normal distribution was tested with the Kolmogorov-Smirnov test, and the Levene's test was used for homogeneity of variance. Based on variable types and distributions, descriptive statistics were used to express continuous variables as median (interquartile range, IQR) or mean (± standard deviation, SD) and dichotomous variables as number (percentage). Differences among the 3 study groups were tested with Pearson's χ^2 test, Fisher-Freeman-Halton test, one-way ANOVA, or Kruskal-Wallis test, as appropriate; with post-hoc pairwise comparisons adjusted by Bonferroni correction.

Surgical outcomes were analyzed for the 2-year follow-up. Two paired-sample *t* test were calculated to determine within-group difference in change of follow-ups with baseline. To determine the clinical relevance of the responses and considering potential differences in baseline assessments for different study groups, we calculated relative changes analyses as percentages of improvement in outcome measures ([follow-up score - baseline score]/baseline score × 100%, for scales with higher scores indicating severer symptoms, such as MDS-UPDRS; or its inverse for scales with higher scores indicating severer symptoms, such as MMSE) and compared between-group difference in relative changes. Correlations were tested by Pearson/Spearman correlation analysis.

In exploring potential surgical prognostic factors, we further applied multivariable linear regression analysis for the primary outcomes. Variables with $P < 0.10$ in the univariable linear regression, which might convey important information, were then entered into the multivariable linear regression (backward elimination) to determine their optimal combination and to adjust for confounding effects. The multicollinearity diagnostics were performed and excluded correlated variables (variance inflation factors, VIF ≥10) from the final model. The multivariable adjusted β coefficient and its 95% confidence interval (CI) were reported for the independent factors.

All the statistical tests were two-sided, and a *P*-value (or Bonferroni adjusted *P*-value) < 0.05 was considered statistically significant. All statistical analyses and figures were performed and drawn using SPSS software, version as used (IBM Corp); R software, version as used (R Foundation); and GraphPad Prism, version as used (GraphPad Software LLC).

Version History

V1.0; January 5th, 2023; First version

V1.1; February 16th, 2023; Added "Surgical outcomes were analyzed for the 2-year follow-up."

V1.2; March 10th, 2023; Approved by the reviewers as the *final version*.

TIME STAMP: March 10th, 2023, Version V1.2
